# Comparative efficacy of commercial Chinese polyherbal preparation for coronary microvascular dysfunction: a systematic review and network meta-analysis of randomized controlled trials

**DOI:** 10.3389/fphar.2025.1642864

**Published:** 2025-11-04

**Authors:** Wujiao Wang, Jun Zhang, Xinyue Wang, Yuxuan Li, Yudou Li, Fenglan Pu, Zhifei Yang, Jie Wan, Haiyan Zhu, Tianli Li, Peifen Chang

**Affiliations:** ^1^ Dongzhimen Hospital of Beijing University of Chinese Medicine, Beijing, China; ^2^ Department of Cardiovascular, Ordos Hospital of Traditional Chinese Medicine, Ordos, China; ^3^ Beijing University of Chinese Medicine, Beijing, China; ^4^ Institute of Basic Theory of Traditional Chinese Medicine, China Academy of Chinese Medical Sciences, Beijing, China; ^5^ National Integrated Traditional and biomedicine Medicine Center for Cardiovascular Disease, China-Japan Friendship Hospital, Beijing, China

**Keywords:** commercial Chinese polyherbal preparation, coronary microvascular dysfunction, frequentist framework, network meta-analysis, randomized controlled trials

## Abstract

**Background:**

Commercial Chinese polyherbal preparations (CCPPs) are widely used in China to treat coronary microvascular dysfunction (CMD). However, the discussion on the best CCPPs continues. This network meta-analysis (NMA) aimed to evaluate and rank the relative efficacy of CCPPs for CMD and summarize the possible mechanisms according to experimental researches.

**Method:**

From the time the database was established to 12 December 2024, We systematically searched eight databases and two registry systems, including Web of Science, Cochrane Library, PubMed, Embase, China National Knowledge Infrastructure (CNKI), Wanfang database, China Science and Technology Journal Database (VIP), Chinese Biomedical Literature database (CBM), Clinical Trials, and the China Clinical Trials Registry. Clinical randomized controlled trials (RCTs) of nine CCPPs in treating CMD, including Shexiangbaoxin Pill (SXBX), Tongxinluo Capsule (TXL), Shexiangtongxindi Pill (SXTXD), Yindanxinnaotong Capsule (YDXNT), Kedalin Tablet (KDL), Xinbao Pill (XB), Xinkeshu Tablet (XKS), Diaoxinxuekang Capsule (DAXXK), and Yixintongluo Capsule (YXTL), were retrieved. The primary outcomes were the Index of Microcirculatory Resistance (IMR) and Coronary Flow Reserve (CFR). Secondary outcomes included the Angina attack frequency, hypersensitive C-reactive protein (hs-CRP), Endothelin-1 (ET-1), Nitric oxide (NO), and Low-density lipoprotein cholesterol (LDL-C). Two researchers performed rigorous data extraction and quality assessment. The quality of the included RCTs was evaluated using the Cochrane Risk of Bias assessment tool, version 2.0 (RoB 2). We then conducted the NMA using a random-effects model under the frequentist framework with Stata version 15. Interventions were ranked based on the surface under the cumulative ranking curve (SUCRA) probability values. The risk of bias was detected using funnel plots and Egger’s test.

**Result:**

A total of 39 RCTs involving 3,240 patients were included in this study. NMA results showed that SXBX had the highest probability of being the best treatment on account of the reduction of IMR [MD = −5.93, 95% CI (−8.75, −3.11)] and LDL-C [[MD = −0.56, 95% CI (−0.99, −0.14)], XB showed better efficacy in improving CFR [MD = 0.71, 95% CI (0.53, 0.89)], TXL showed better efficacy in angina attack frequency [MD = −5.30, 95% CI (−7.08, −3.53)]; YXTL showed better efficacy in hs-CRP [MD = −5.04, 95% CI (−8.38, −1.7)]; XKS showed better efficacy in ET-1 [MD = −43.3, 95% CI (−59.71, −26.89)]; YDXNT showed better efficacy in NO [MD = 17.69, 95% CI (6.07, 29.32)]. In addition, the protective effect of CCPP on CMD may be achieved by altering multiple signalling pathways through anti-atherosclerosis, anti-vascular smooth muscle cell proliferation and migration, anti-inflammation, antioxidant stress, protection of vascular endothelium, improving energy metabolism, antiplatelet activation and aggregation, and promoting angiogenesis.

**Conclusion:**

CCPPs combined with conventional therapy led to a significant improvement in CFR and NO, as well as a reduction in IMR, angina attack frequency, hs-CRP, ET-1, and LDL-C levels. SXBX emerged as the optimal treatment regimen for lowering IMR and LDL-C levels. Additionally, XB demonstrated superiority in improving CFR. TXL demonstrated superiority in reducing angina attack frequency, YXTL in lowering hs-CRP levels, XKS in lowering ET-1 levels, and YDXNT in increasing NO levels. Nevertheless, the majority of the evidence was rated as low certainty according to the GRADE assessment. Conclusion should be framed as hypothesis-generating rather than definitive, and there is a need for large-scale, multicenter, and direct comparative RCTs of CCPPs treated for CMD to generate higher-quality evidence.

**Systematic review registration:**

https://www.crd.york.ac.uk/PROSPERO/, identifier CRD42025632143.

## 1 Introduction

Coronary microvascular dysfunction (CMD) is a phenomenon in which the coronary microcirculation is structurally and/or functionally altered, causing impaired coronary blood flow and ultimately leading to myocardial ischemia (Del Buono et al., 2021). CMD is highly prevalent — affecting >50% of patients with diabetes mellitus and 70%–85% of those with heart failure with preserved ejection fraction (Aljizeeri et al., 2023; Arnold et al., 2022; Ford et al., 2018), and present in 45%–60% of patients with non-obstructive coronary artery disease (CAD) (Rehan et al., 2023). CMD is most commonly seen in symptomatic patients with chronic coronary syndromes and recurrent angina pectoris at rest or on exertion despite the absence of obstructive CAD (Samuels et al., 2023; Dimitriadis et al., 2024). Studies have shown that in the absence of epicardial coronary artery disease, the frequency of angina episodes in patients with CMD can be as high as 1–3 episodes/week (Ford et al., 2018), which seriously affects their quality of life. In addition, patients with CMD had a 3.93-fold increase in total mortality and a 5.16-fold increase in adverse cardiovascular events compared with those with normal coronary microcirculation (Gdowski et al., 2020). Impaired coronary flow reserve (CFR) and novel indices, such as microvascular resistance reserve, are strong, independent predictors of adverse cardiovascular outcomes (Kelshiker et al., 2022; Dimitriadis et al., 2025).

Empirical treatment of CMD is based on traditional therapies for CAD, including antiplatelet, lipid-lowering, and anti-ischemic therapy (e.g., nitrates, beta-blockers, angiotensin-converting enzyme inhibitors) (Camici and Crea, 2007; Crea et al., 2014). However, the curative effect of experiential therapy alone on CMD is not evident. Multiple novel drugs that primarily reduce angina, including ranolazine, ivabradine, nicorandil, and zibotentan, have been evaluated in patients with CMD. Two meta-analyses (Zhuet al., 2019; Khandkar et al., 2025) showed that, compared with the control group, Ranolazine, Nicorandil, and Ivabradine did not improve the CFR. A recent RCT (Morrow et al., 2024) showed that short-term zibotentan treatment did not show any benefits for CMD. The number of patients with coronary microcirculatory disorders in clinical practice is large, the mechanism is complex, and the effect of conventional drug therapy is still unsatisfactory. Therefore, looking for potential complementary and alternative therapies for this significant medical need is essential.

The use of complementary and alternative medicine therapies in the treatment of CMD has received much attention in recent years. In China, as one of the primary intervention measures, Traditional Chinese Medicine (TCM) has gradually developed a scientific approach to compatibility and an industrialized production process over time, resulting in commercial Chinese polyherbal preparations (CCPP) (Zhang et al., 2024). CCPPs have been included in the Chinese Pharmacopoeia and have apparent efficacy and indications. They are a key metabolite in the Chinese pharmaceutical market. Compared with TCM decoctions, CCPPs have the advantages of stable quality, a good curative effect, good safety, fast absorption, convenience in taking, carrying, and storage (Hsu E., 2009; Kang et al., 2019). In addition to being an adjunctive therapy, it can also serve as an alternative treatment option in resource-limited settings or for investigational purposes. CCPPs are widely used as adjunctive therapy for CMD in China. Such as Shexiangbaoxin Pill (SXBX) (Sun, 2021), Tongxinluo Capsule (TXL) (Fang et al., 2018), Shexiangtongxindi Pill (SXTXD) (Liu et al., 2018), Yindanxinnaotong Capsule (YDXNT) (Wang, 2022), Kedalin Tablet (KDL) (Lin et al., 2023), Xinbao Pill (XB) (Zhang, 2022), Xinkeshu Tablet (XKS) (Chen et al., 2019), Diaoxinxuekang Capsule (DAXXK) (Wang B. et al., 2024), and Yixintongluo Capsule (YXTL) (Meng, 2019), whose efficacy in increasing CFR, improving clinical symptoms of angina, Reducing inflammatory response and improving vascular endothelial function have been recognized. Therefore, this study conducted a network meta-analysis (NMA) of randomized controlled trials (RCTs) on nine CCPPs for the treatment of CMD. The aim was to comprehensively evaluate and rank the relative potential for CMD of CCPPs among all available publications.

## 2 Materials and methods

### 2.1 Registration and reporting

This NMA was conducted under the Preferred Reporting Items for Systematic Reviews and Meta-Analysis extension statement for network meta-analysis (PRISMA-NMA) (Hutton et al., 2015). This study was registered with PROSPERO under registration number CRD42025632143.

### 2.2 Standard evaluation of CCPPs

To enhance the accuracy, the CCPPs in this study were reported in accordance with the requirements of the Consensus statement on the Phytochemical Characterisation of Medicinal Plant extracts (ConPhyMP) (Heinrich et al., 2022). Accurate scientific nomenclature for botanical drugs referred to Rivera’s suggestion (Rivera et al., 2014) and was validated taxonomically in the databases of “Medicinal Plant Names Services” (https://mpns.science.kew.org/mpns-portal/). The composition and standardised name for each CCPP were presented in Table 1. In addition, we referred to the Chinese Pharmacopoeia 2025 regarding the names of non-botanical drugs. The relevant information about CCPPs referred to the original study, the Chinese Pharmacopoeia 2025, and the National Medical Products Administration. The details were shown in Supplementary Appendix S2, S3.

**TABLE 1:** Composition of CCPPs.

CCPPs	Source	Constituent(s)	Usage and dosage (medicine instruction)	Quality control reported?	Chemical analysis reported? (Y/NR)
Shexiangbaoxin Pill	Shanghai Hehuang Pharmaceutical Co., Ltd	Moschus* [Moschidae; *Moschus berezovskii* Flerov, dried secretion], Ginseng Radix et Rhizoma [Araliaceae; *Panax ginseng* C.A.Mey., root and rhizome], Bovis calculus* [Bovidae; *Bos taurus* Linnaeus, gallstone], Cinnamomi Cortex* [Lauraceae; *Cinnamomum verum* J. Presl, bark], Styrax*[Altingiaceae; *Liquidambar orientalis* Mill., purified balsam], Bufonis Venenum* [Bufonidae; *Bufo bufo gargarizans* Cantor, dried secretion], Borneolum* [Lauraceae; *Cinnamomum camphora* (L.) J. Presl, synthetic product]	1–2 pills (22.5mg/pill), tid, po	Y-Prepared according to NMPA: Z31020068	NR
Tongxinluo Capsule	Shijiazhuang Ealing Pharmaceutical Co., Ltd	Ginseng Radix et Rhizoma [Araliaceae; *Panax ginseng* C.A.Mey, root and rhizome], Scorpio* [Buthidae; *Buthus martensii* Karsch, dried body], Hirudo* [Hirudinidae; *Hirudo nipponica* Whitman, dried body], Eupolyphaga/Steleophaga* [Corydiidae; *Eupolyphaga sinensis* Walker, dried female body],Scolopendra* [Scolopendridae; *Scolopendra subspinipes mutilans* L. Koch, dried body], Cicadae Periostracum* [Cicadidae; *Cryptotympana pustulata* Fabricius, nymph exuviae], Paeoniae Radix Alba [Paeoniaceae; *Paeonia lactiflora* Pall., root], Borneolum* [Lauraceae; Synthetic product derived from *Cinnamomum camphora* (L.) J. Presl], Santalum Albi Lignum [Santalaceae; *Santalum album* L., heartwood], Dalbergiae Odoriferae Lignum [Fabaceae; *Dalbergia odorifera* T.C. Chen, heartwood], Olibanum [Burseraceae; *Boswellia carterii* Birdw., resin], Ziziphi Spinosae Semen [Rhamnaceae; *Ziziphus jujuba* var*. spinosa* (Bunge) Hu exH. F. Chow, seed]	2–4 capsules (0.26g/capsule), tid, po	Y-Prepared according to NMPA: Z19980015	NR
Shexiangtongxindi Pill	InnerMongolia Kang En Bei Pharmaceutical Co., Ltd	Moschus* [Moschidae; *Moschus berezovskii* Flerov, dried secretion], Ginseng Radix et Rhizoma [Araliaceae; *Panax ginseng* C. A. Mey., root and rhizome], Bufonis Venenum* [Bufonidae; *Bufo bufo gargarizans* Cantor, dried secretion], Salviae Miltiorrhizae Radix et Rhizoma [Lamiaceae; *Salvia miltiorrhiza* Bunge, rootand rhizome], Bovis Calculus* [Bovidae; *Bos taurus* Linnaeus, gallstone], Fel Ursi* [Ursidae; *Ursus thibetanus* Cuvier and/or *Ursus arctos* Linnaeus, gallbladder. The original trial report lacked specificationof the exact species], Borneolum* [Lauraceae; Synthetic productderived from *Cinnamomum camphora* (L.) J. Presl. The exact source material was not detailed in the report]	2 pills (35mg/pill), tid, po	Y-Prepared according to NMPA: Z20080018	NR
Yindanxinnaotong Capsule	Guizhou Bailing Enterprise Group Pharmaceutical Co., Ltd	Ginkgo Folium [Ginkgoaceae; *Ginkgo biloba* L., leaf], Salviae Miltiorrhizae Radix et Rhizoma [Lamiaceae; *Salvia miltiorrhiza* Bunge, root and rhizome], Erigerontis Herba [Asteraceae; *Erigeron breviscapus* (Vaniot) Hand.-Mazz., whole herb], Gynostemmatis Herba [Cucurbitaceae; *Gynostemma pentaphyllum* (Thunb.) Makino, whole plant], Crataegi Fructus [Rosaceae; *Crataegus pinnatifida* Bunge, fruit], Allii Sativi Bulbus [Liliaceae; *Alium sativum* L., bulb], Notoginseng Radix et Rhizoma [Araliaceae; *Panax notoginseng* (Burkill) F. H. Chen, root and rhizome], Blumeae Folium [Asteraceae; *Blumea balsamifera* (L.) DC., leaf]	2–4 capsules (0.4g/capsule), tid, po	Y-Prepared according to NMPA: Z20027144	NR
Kedalin Tablet	Zhejiang Kang Enbei Pharmaceutical Co., Ltd	Corydalis Rhizoma [Papaveraceae; *Corydalis yanhusuo* W. T. Wang, rhizome]	2–3 tablets (2.4mg/capsule), tid, po	Y-Prepared according to NMPA: Z20044361	NR
Xinbao Pill	Guangdong Xinbao Pharmaceutical Technology Co., Ltd	Daturae Flos [Solanaceae; *Datura mete*/L, dried flower], Ginseng Radix et Rhizoma [Araliaceae; *Panax ginseng* C. A. Mey., root and rhizome], Cinnamomi Cortex [Lauraceae; *Cinnamomum verum* J. Presl, bark], Aconiti Lateralis Radix Praeparata [Ranunculaceae; *Aconitum carmichaelii* Debx., processed lateral root], Cervi Cornu Pantotrichum* [Cervidae; *Cervus nippon* Temminck and/or *Cervus elaphus* Linnaeus, unossified antler. The original source did not specify which species was used.], Borneolum* [Lauraceae; Synthetic borneolderived from *Cinnamomum camphora* (L.) J. Presl. The source material (fresh branches and leaves)is inferred from common preparation methods], Moschus* [Moschidae; *Moschus berezovskii* Flerov, dried secretion], Notoginseng Radix et Rhizoma [Araliaceae; *Panax notoginseng* (Burkill) F. H. Chen, root and rhizome], Bufonis Venenum* [Bufonidae; *Bufo bufogargarizans* Cantor, dried secretion]	2–6 pills (60mg/pill), tid, po	Y-Prepared according to NMPA: Z44021843	NR
Xinkeshu Tablet	Shandong Wohua Pharmaceutical Technology Co., Ltd	Salviae Miltiorrhizae Radix et Rhizoma [Lamiaceae; *Salvia miltiorrhiza* Bunge, root and rhizome], Puerariae Lobatae Radix [Fabaceae (Leguminosae); *Pueraria lobata* (Willd.) Ohwi, root], Notoginseng Radix et Rhizoma [Araliaceae; *Panax notoginseng* (Burkill) F. H. Chen, root and rhizome], Crataegi Fructus [Rosaceae; *Crataegus pinnatifida* Bunge, fruit], Aucklandiae Radix [Asteraceae; *Aucklandia lappa* Decne., root]	4 tablets (0.31g/capsule), tid, po	Y-Prepared according to NMPA: Z37020042	NR
Diaoxinxuekang Capsule	Chengdu Dio Pharmaceutical Group Co., Ltd	Dioscoreae Rhizoma [Dioscoreaceae; *Dioscorea panthaica* Prain et Burk. and/or *Dioscorea nipponica* Makino, rhizome. The original trial report did not specify the exact species used]	1–2 capsules (0.1g/capsule), tid, po	Y-Prepared according to NMPA: Z20050616	NR
Yixintongluo Capsule	Lu Pharmaceutical Co., Ltd	Astragali Radix [Fabaceae; *Astragalus membranaceus* (Fisch.) Bunge, root], Ginseng Radix et Rhizoma [Araliaceae; *Panax ginseng* C. A. Mey., root and rhizome], Ophiopogonis Radix [Asparagaceae; *Ophiopogon japonicus* (L. f.) Ker Gawl., root], Salviae Miltiorrhizae Radix et Rhizoma [Lamiaceae; *Salvia miltiorrhiza* Bunge, root and rhizome], Dalbergiae Odoriferae Lignum [Fabaceae; *Dalbergia odorifera* T. C. Chen, heartwood], Aurantii Fructus [Rutaceae; *Citrus aurantium* L., fruit], Chuanxiong Rhizoma [Apiaceae; *Ligusticum chuanxiong* Hort., rhizome], Poria [Polyporaceae; *Wolfiporia cocos* (F. A.Wolf) Ryvarden et Gilb., sclerotium], Pinelliae Rhizoma [Araceae; *Pinellia ternata* (Thunb.) Makino, tuber], Trichosanthis Pericarpium [Cucurbitaceae; *Trichosanthes kirilowii* Maxim., pericarp], Allii Macrostemonis Bulbus [Amaryllidaceae; *Allium macrostemon* Bunge, bulb], Citri Reticulatae Pericarpium [Rutaceae; *Citrus reticulata* Blanco, pericarp], Glycyrrhizae Radix et Rhizoma [Fabaceae; *Glycyrrhizaa uralensis* Fisch. ex DC., root and rhizome]	4 capsules (NR/capsule), tid, po	Y-Prepared according to NMPA: ZBZ1789	NR

### 2.3 Search strategy

From the time the database was established to 12 December 2024, eight databases and two registry systems were searched, including Web of Science, Cochrane Library, PubMed, Embase, China National Knowledge Infrastructure (CNKI), Wanfang database, China Science and Technology Journal Database (VIP), Chinese Biomedical Literature database (CBM), Clinical Trials, and the China Clinical Trials Registry. Clinical RCTs of nine CCPPs in treating CMD, including SXBX, TXL, SXTXD, YDXNT, KDL, XB, XKS, DAXXK, and YXTL were retrieved. Additionally, we manually searched references of eligible studies to identify other relevant research. The search is elaborated further in the Supplemental appendix 4, encompassing additional search strategies and outcomes information.

### 2.4 Study selection

The Population-Intervention-Comparators-Outcomes-Timing-Setting (PICOTS) framework was used as the criterion for this study. Inclusion criteria: (1) Population: All patients were diagnosed with CMD. (2) Intervention: Conventional therapy combined with SXBX or TXL or SXTXD or YDXNT or KDL or XB or XKS or DAXXK or YXTL. (3) Comparator: Conventional therapy. (4) Outcomes: The primary outcome indicators were the IMR and CFR; The Secondary outcome measures were Angina attack frequency, hs-CRP, ET-1, NO, and LDL-C. (5) Timing: Studies with any follow-up duration were considered. (6) Setting: Studies conducted in any clinical setting (e.g., inpatient, outpatient) were eligible (7) Study design: RCTs.

Exclusion criteria: (1) Non-RCT. (2) The intervention was a combination of multiple therapies or did not specify a therapeutic agent. (3) Duplicate publication. (4) Retracted. (5) Full text unavailable. (6) Lack of complete data.

### 2.5 Data extraction

Two independent reviewers (Yudou Li and Xinyue Wang) extracted the following details from the included studies: (1) first author’s name, year, and country of publication; (2) sample size and mean age; (3) specific interventions, duration of interventions; and (4) outcome data. Any disagreements were discussed or consulted with the third researcher (Wujiao Wang).

### 2.6 Risk of bias

The quality of the included studies was evaluated using the Cochrane Collaboration’s risk of bias assessment tool, version 2.0 (RoB 2). The quality assessment items were as follows: (1) Randomization process; (2) Deviations from intended interventions; (3) Missing outcome data; (4) Measurement of the outcome; (5) Selection of the reported result; and (6) Overall risk of bias. Bias in each aspect was evaluated as “low risk,” “some concerns,” and “high risk.” Any disagreements were discussed or consulted with the third researcher (Peifen Chang).

### 2.7 Statistical analysis

First, Risk ratios (RRs) and 95% CI were performed for dichotomous variables, and mean difference (MD) and 95% CI were performed for continuous variables. In certain multi-arm trials (such as three-arm studies), if two control arms both qualify as active controls, they are merged into a single active control group using established formulas (Supplementary Appendix S5), in order to prevent duplication of experimental group data and inflated contribution to the overall analysis. Data were analysed using a random-effects model under the frequentist framework with Stata version 15. Network diagrams were constructed to visualise the geometry of the treatment network. In these diagrams, node size is proportional to the total sample size for each treatment, and the thickness of the connecting lines represents the number of studies that directly compared the connected interventions. Provided that the closed loop of interventions was available, Global consistency was evaluated using the design-by-treatment interaction model, while local inconsistency was examined using node-splitting analysis, which compares direct and indirect evidence for specific treatment comparisons. Between-study heterogeneity was quantified by estimating the variance (τ^2^) of the underlying effect sizes, with parameters estimated using the restricted maximum likelihood method. Meta-regression analysis is employed to investigate the potential influence of covariates on intervention effect estimates. Sensitivity analysis is utilised to assess the robustness. Then, interventions were ranked using the Surface Under the Cumulative Ranking Curve (SUCRA) probability values, with higher SUCRA values indicating a greater likelihood of a treatment being ranked highly. Finally, the Funnel plots and Egger’s test were used to explore publication bias if >10 studies were included.

### 2.8 Grading of the evidence

The quality of evidence was assessed using the GRADE method (Guyatt et al., 2011). It was categorized as high, moderate, low, or very low. RCTs received a high initial grade by default and were downgraded according to pre-specified criteria: risk of bias, inconsistency, indirectness, imprecision, and other considerations.

## 3 Result

### 3.1 Study selection

**FIGURE 1:**
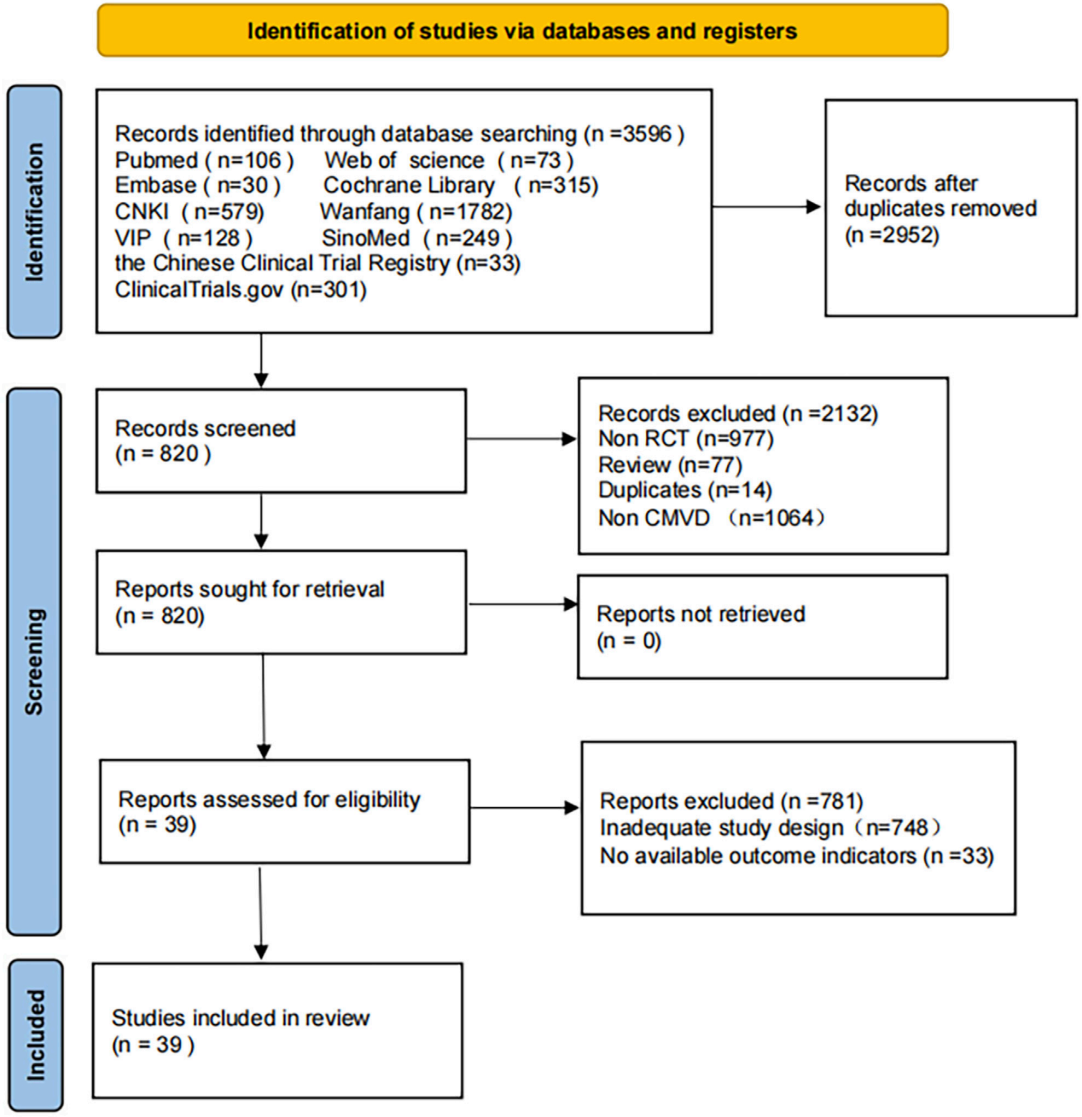
Flow diagram of the literature searches.

The preliminary search obtained 3,596 relevant papers, 2,952 papers were obtained after excluding duplicates, and 820 papers were obtained after excluding Non-RCTs, Reviews, Non-CMD, and duplicates. Thirty-nine papers (Bai et al., 2022; Chen et al., 2022; Chen et al., 2019; Chu, 2021; Fang et al., 2018; Feng et al., 2005; Fu, 2021; Fu et al., 2020; Gong et al., 2021; Jiang et al., 2024; Li, 2021; Li and Tang, 2009; Liang et al., 2019; Lin et al., 2023; Liu et al., 2003; Liu et al., 2018; Lv et al., 2014; Ma and Xu, 2006; Meng, 2018; 2019; Peng, 2011; Qi et al., 2023; Qin et al., 2017; Qin et al., 2023; Ren et al., 2023; Shen et al., 2021; Sun et al., 2022; Sun, 2021; Wang B. et al., 2024; Wang, 2022; Wang, 2015; Wang and Long, 2022; Wang et al., 2019; Wei, 2018; Wu et al., 2019; Xie et al., 2019; Zhang, 2022; Zhang et al., 2013; Zhao D. H. et al., 2021) were finally included after further reading the full text to exclude studies with Inadequate study design and unavailable research data. Tianli Li resolved any disagreements during the selection process. The Flow diagram of the literature search is shown in Figure 1.

### 3.2 Characteristics of included studies

A total of 39 studies were included, all of which were published in Chinese. There were 3,240 participants, including 1,622 in the treatment group and 1,618 in the control group. Detailed characteristics of the included studies are shown in Table 2.

**TABLE 2:** General characteristics of the studies.

Study	Number of participants		Age (years)		Gender (M/F)	CMD diagnostic criteria	Interventions		Treatment duration	Outcome index	P-value
	Intervention	Control	Intervention	Control			Intervention	Control			
Qi et al. (2023)	53	55	46.5 ± 1.7	42.8 ± 1.7	73/35	Single-photon emission computed tomography (SPECT), myocardial perfusion imaging (transient ischaemic dilation TID >1.2)	SXBX + CT	CT	12w	1. ET-1 2. NO 3. hs-CRP 4. LDL-C	*1. P* < 0.05 2. *P* < 0.05 3. *P* < 0.05 4. *P* < 0.05
Wang, HZ. (2015)	20	20	58 ± 3	59 ± 4	15/25	With typical angina pectoris symptoms and electrocardiographic evidence of ischaemic ST-T changes, with normal coronary angiography	SXBX + CT	CT	8w	1. Angina attack frequency	1. *P* < 0.05
Qin et al. (2017)	30	30	46.9 ± 6.2	47.2 ± 2.2	24/36	With typical angina pectoris symptoms and electrocardiographic evidence of ischaemic ST-T changes, with normal coronary angiography	SXBX + CT	CT	8w	1. Angina attack frequency	1. *P* < 0.05
Sun (2021)	43	43	72.84 ± 6.36	74.93 ± 7.88	34/52	NR	SXBX + CT	CT	6w	1. Angina attack frequency 2. LDL-C	1. *P* < 0.05 2. *P* < 0.05
Wang, XM. (2022)	40	40	53.52 ± 2.24	53.55 ± 2.21	47/33	NR	SXBX + CT	CT	12w	1. Angina attack frequency 2. LDL-C	1. *P* < 0.05 2. *P* < 0.05
Sun et al. (2022)	56	55	51.23 ± 13.85	52.60 ± 14.76	68/43	Coronary angiography indicates a stenosis of less than 50% in the diameter of the epicardial coronary artery, Myocardial radionuclide imaging indicates myocardial ischaemia	SXBX + CT	CT	4w	1. ET-1 2. NO	1. *P* < 0.05 2*. P* < 0.05
Shen et al. (2021)	32	32	41.38 ± 9.43	45.75 ± 10.61	38/26	Coronary angiography indicates a stenosis of less than 50% in the diameter of the epicardial coronary artery, IMR>32	SXBX + CT	CT	48w	1. IMR 2. LDL-C	1. *P* < 0.05 2. *P* > 0.05
Wu et al. (2019)	38	38	53.7 ± 2.6	54.1 ± 2.5	47/29	Coronary angiography indicates a stenosis of less than 50% in the diameter of the epicardial coronary artery, TIMI Frame Count >27	SXBX + CT	CT	12w	1. ET-1 2. NO 3. hs-CRP	1. *P* < 0.05 2. *P* < 0.05 3*. P* < 0.05
Chu, YX. (2021)	37	37	51.4 ± 10.5	51.6 ± 10.3	41/33	NR	SXBX + CT	CT	4w	1. Angina attack frequency	1. *P* < 0.05
Bai et al. (2022)	39	39	62.71 ± 7.24	64.55 ± 6.14	38/40	NR	SXBX + CT	CT	12w	1. IMR 2. ET-1 3. NO	1. *P* < 0.01 2. *P* < 0.01 3*. P* < 0.01
Fu, ZH. (2021)	82	82	61.48 ± 12.49	62.13 ± 11.57	106/58	NR	SXBX + CT	CT	8w	1. NO 2. hs-CRP	1. *P* < 0.05 2. *P* < 0.05
Zhang et al. (2013)	28	28	NR	NR	23/33	With typical angina pectoris symptoms and positive ECG treadmill exercise test, with normal coronary angiography	SXBX + CT	CT	6w	1. Angina attack frequency2. LDL-C	1. *P* < 0.05 2. *P* < 0.05
Chen et al. (2022)	35	35	56.0 ± 9.2	59.0 ± 9.8	42/28	With typical angina pectoris symptoms and positive ECG treadmill exercise test, with normal coronary angiography	SXBX + CT	CT	4w	1. Angina attack frequency 2. ET-1 3. NO	1. *P* < 0.05 2. *P* < 0.05 3*. P* < 0.05
Wei, Y. (2018)	30	32	46.5 ± 3.3	46.3 ± 3.2	0/62	Coronary angiography indicates a stenosis of less than 20% in the diameter of the epicardial coronary artery,CFR<2	TXL + CT	CT	12w	1. ET-1 2. NO	1*. P* < 0.05 2. *P* < 0.05
Ma and Xu (2006)	24	20	50.1 ± 7.0	48.8 ± 6.2	NR	With typical angina pectoris symptoms and positive ECG treadmill exercise test, Coronary angiography indicates a stenosis of less than 50% in the diameter of the epicardial coronary artery	TXL + CT	CT	12w	1. ET-1 2. NO	1*. P* < 0.05 2. *P* < 0.05
Fang et al. (2018)	34	34	45.7 ± 6.9	42.2 ± 6.0	31/37	With typical angina pectoris symptoms and positive ECG treadmill exercise test, Coronary angiography indicates a stenosis of less than 50% in the diameter of the epicardial coronary artery	TXL + CT	CT	12w	1. Angina attack frequency 2. ET-1 3. NO	1*. P* < 0.05 2. *P* < 0.05 3. *P* < 0.05
Feng et al. (2005)	16	16	42.2 ± 7.51	41.6 ± 6.99	15/17	With typical angina pectoris symptoms and positive ECG treadmill exercise test, Coronary angiography indicates a stenosis of less than 50% in the diameter of the epicardial coronary artery	TXL + CT	CT	4w	1. Angina attack frequency 2. ET-1	1. *P* < 0.01 2. *P* < 0.01
Xie et al. (2019)	45	45	58.6 ± 7.8	58.8 ± 8.2	41/49	NR	TXL + CT	CT	4w	1. LDL-C 2. hs-CRP	1. *P* < 0.01 2. *P* < 0.01
Li and Tang (2009)	36	32	58.2 ± 9.5	56.7 ± 8.2	30/38	With typical angina pectoris symptoms and positive ECG treadmill exercise test, Coronary angiography indicates a stenosis of less than 50% in the diameter of the epicardial coronary artery	TXL + CT	CT	4w	1. LDL-C 2. ET-1 3. NO	1*. P* < 0.01 2. *P* < 0.01 3. *P* < 0.01
Jiang et al. (2024)	106	106	62.88 ± 2.01	62.47 ± 2.31	100/112	NR	TXL + CT	CT	12w	1. ET-1	1. *P* < 0.01
Liu et al. (2003)	19	18	NR	NR	NR	With typical angina pectoris symptoms and positive ECG treadmill exercise test, Coronary angiography indicates a stenosis of less than 50% in the diameter of the epicardial coronary artery	TXL + CT	CT	8w	1. ET-1	1. *P* < 0.01
Lv et al. (2014)	19	19	NR	NR	12/26	With typical angina pectoris symptoms and positive ECG treadmill exercise test, Coronary angiography indicates a stenosis of less than 50% in the diameter of the epicardial coronary artery	TXL + CT	placebo + CT	12w	1. ET-1 2. NO	1*. P* < 0.01 2. *P* < 0.01
Gong et al. (2021)	54	52	62.71 ± 5.32	61.98 ± 5.39	47/59	With typical angina pectoris symptoms, Coronary angiography indicates a stenosis of less than 50% in the diameter of the epicardial coronary artery, positive ECG treadmill exercise test or CFR<2	SXTXD + CT	CT	12w	1. ET-1 2. NO 3. hs-CRP	1*. P* < 0.05 2. *P* < 0.05 3. *P* < 0.05
Liu et al. (2018)	20	18	51.00 ± 8.45	51.95 ± 8.48	12/26	With typical angina pectoris symptoms and positive ECG treadmill exercise test, Coronary angiography indicates a stenosis of less than 50% in the diameter of the epicardial coronary artery	SXTXD + CT	CT	12w	1. Angina attack frequency2. hs-CRP	1*. P* < 0.05 2. *P* < 0.05
Qin et al. (2023)	55	56	55.78 ± 8.55	57.00 ± 10.00	63/48	With typical angina pectoris symptoms, Coronary angiography indicates a stenosis of less than 50% in the diameter of the epicardial coronary artery, CFR<2	SXTXD + CT	CT	24w	1. CFR	1*. P* < 0.05
Li (2021)	36	36	61.23 ± 6.37	60.92 ± 6.14	37/35	Meets the clinical diagnostic criteria for coronary artery slow blood flow, IMR>25	SXTXD + CT	CT	24w	1. IMR	1*. P* < 0.05
Peng (2011)	23	23	NR	NR	18/28	With typical angina pectoris symptoms and positive ECG treadmill exercise test, Coronary angiography indicates a stenosis of less than 50% in the diameter of the epicardial coronary artery	YDXNT + CT	CT	8w	1. Angina attack frequency	1*. P* < 0.05
Wang (2022)	65	65	57.82 ± 4.79	58.17 ± 3.36	58/72	NR	YDXNT + CT	CT	24w	1. ET-1 2. NO	1*. P* < 0.01 2. *P* < 0.01
Wang et al. (2019)	43	44	57.3 ± 11.9	56.1 ± 13.2	46/41	With typical angina pectoris symptoms and electrocardiographic evidence of ischaemic ST-T changes, Coronary angiography indicates a stenosis of less than 20% in the diameter of the epicardial coronary artery, CFR< 2,One of the three main coronary arteries exhibits a TIMI flow grade exceeding 27 frames	YDXNT + CT	CT	24w	1.IMR 2. ET-1 3. NO 4. hs-CRP	1. *P* < 0.01 2. *P* < 0.01 3. *P* < 0.01 4. *P* < 0.01
Lin et al. (2023)	46	48	55.6 ± 10.4	58.3 ± 11.6	61/33	With typical angina pectoris symptoms and electrocardiographic evidence of ischaemic ST-T changes, Coronary angiography indicates a stenosis of less than 50%–70% in the diameter of the epicardial coronary artery, IMR>25	KDL + CT	CT	24w	1. IMR 2. CFR	1*. P* < 0.05 2. *P* < 0.05
Fu et al. (2020)	32	32	44.8 ± 7.3	45.5 ± 6.7	22/42	With typical angina pectoris symptoms and positive ECG treadmill exercise test, Coronary angiography indicates a stenosis of less than 20% in the diameter of the epicardial coronary artery	KDL + CT	CT	12w	1. Angina attack frequency	1*. P* < 0.05
Zhang (2022)	40	40	69.51 ± 2.66	67.21 ± 3.54	43/37	NR	XB + CT	CT	12w	1. CFR 2. Angina attack frequency	1*. P* < 0.05 2. *P* < 0.05
Zhao et al. (2021a)	61	61	58.31 ± 7.34	60.03 ± 6.97	58/64	With typical angina pectoris symptoms and positive ECG treadmill exercise test, with normal coronary angiography	XB + CT	CT	12w	1. CFR 2. Angina attack frequency 3. LDL-C	1. *P* < 0.01 2. *P* < 0.01 3. *P* < 0.01
Ren et al. (2023)	44	44	54.84 ± 6.63	55.03 ± 5.79	48/40	Quantitative Assessment of Myocardial Perfusion Imaging by Nuclear Magnetic Resonance, MPRI<2,IMR>24,Coronary angiography indicates a stenosis of less than 50% in the diameter of the epicardial coronary artery	DAXXK + CT	CT	12w	1. IMR	1*. P* < 0.05
Wang et al. (2024a)	43	44	60.1 ± 10.7	61.0 ± 8.5	32/55	Single-photon emission computed tomography (SPECT) revealed myocardial perfusion insufficiency, Trans-thoracic Doppler echocardiography (TTDE) revealed coronary flow reserve (CFR) < 2.0. Coronary angiography indicates a stenosis of less than 20% in the diameter of the epicardial coronary artery	DAXXK + CT	CT	12w	1. CFR	1*. P* < 0.05
Liang et al. (2019)	38	39	69.97 ± 8.48	70.46 ± 7.75	33/44	With typical angina pectoris symptoms and electrocardiographic evidence of ischaemic ST-T changes, Coronary angiography indicates a stenosis of less than 20% in the diameter of the epicardial coronary artery, CFR<2	XKS + CT	CT	24w	1. hs-CRP	1*. P* < 0.05
Chen et al. (2019)	60	60	66.1 ± 4.6	66.7 ± 3.7	69/51	With typical angina pectoris symptoms and positive ECG treadmill exercise test, Coronary angiography indicates a stenosis of less than 50% in the diameter of the epicardial coronary artery	XKS + CT	CT	24w	1. ET-1 2. NO	1*. P* < 0.05 2. *P* < 0.05
Meng (2018)	60	60	57.47 ± 6.51	58.76 ± 6.17	55/65	NR	YXTL + CT	CT	8w	1. IMR	1*. P* < 0.05
Meng (2019)	40	40	59.33 ± 6.26	60.15 ± 7.03	36/44	IMR>32,TIMI flow grade 2	YXTL + CT	CT	8w	1. IMR 2. ET-1 3. hs-CRP	1. *P* < 0.01 2. *P* < 0.05 3. *P* < 0.01

SXBX, shexiangbaoxin pill; TXL, tongxinluo capsule; SXTXD, shexiangtongxindi pill; YDXNT, yindanxinnaotong capsule; KDL, kedalin tablet; XB, xinbao pill; XKS, xinkeshu tablet; DAXXK, diaoxinxuekang capsule; YXTL, yixintongluo capsule; CT, conventional treatment; w, week.

### 3.3 Network plot

**FIGURE 2:**
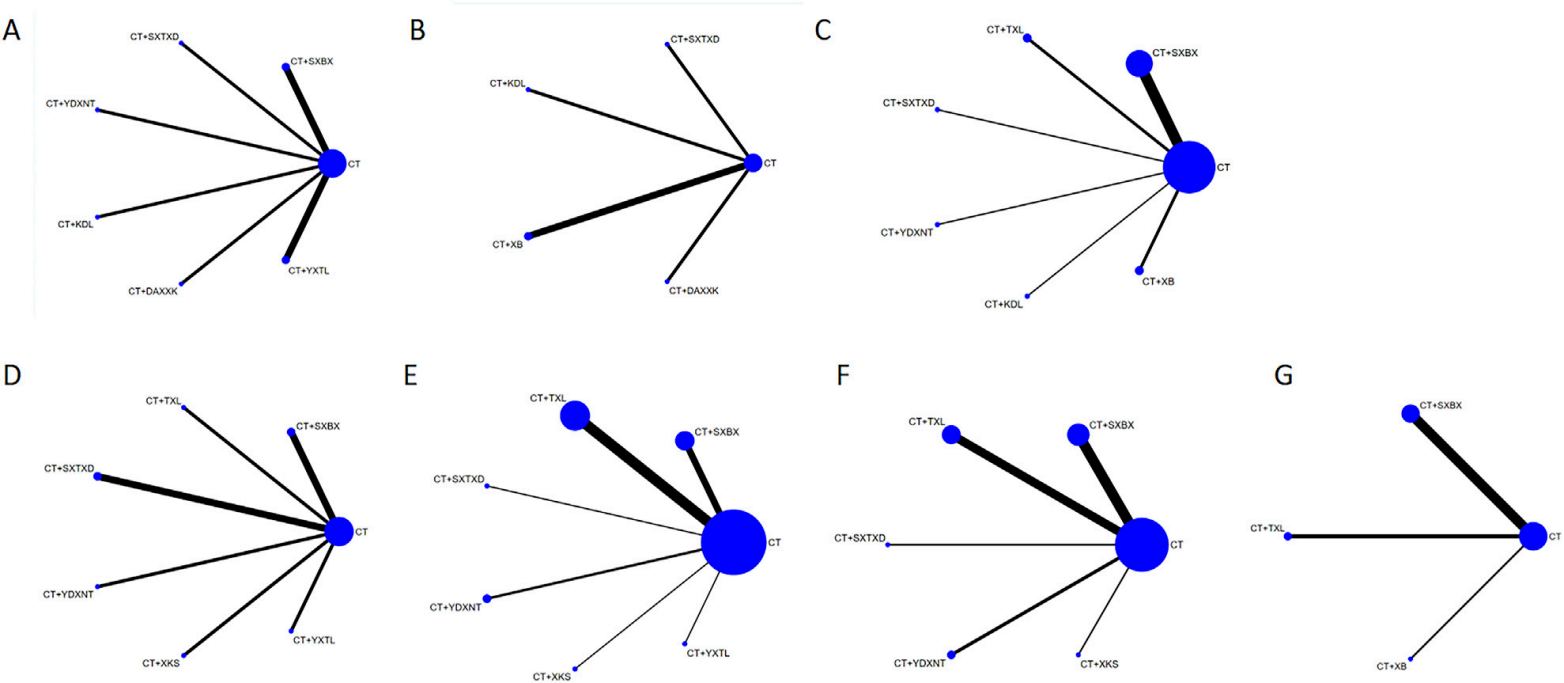
Network graph of the outcomes. **(A)** IMR. **(B)** CFR. **(C)** Angina attack frequency. **(D)** hs-CRP. **(E)** ET-1. **(F)** NO. **(G)** LDL-C. SXBX, Shexiangbaoxin Pill. TXL, Tongxinluo Capsule. SXTXD, Shexiangtongxindi Pill. YDXNT, Yindanxinnaotong Capsule. KDL, Kedalin Tablet. XB, Xinbao Pill. XKS, Xinkeshu Tablet. DAXXK, Diaoxinxuekang Capsule. YXTL, Yixintongluo Capsule. CT, Conventional therapy.

The compared connections among interventions for each outcome are shown in Figure 2. Each node represents a different intervention and the size of nodes is positively correlated with the number of patients. The thickness of the line segment corresponds to the number of included studies for that intervention. The thicker the line segment, the larger the number of included studies for that intervention is. There were no closed loops formed between the studies.

### 3.4 Study quality

**FIGURE 3:**
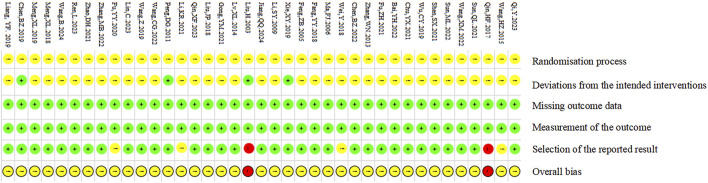
Risk of bias summary.

**FIGURE 4:**
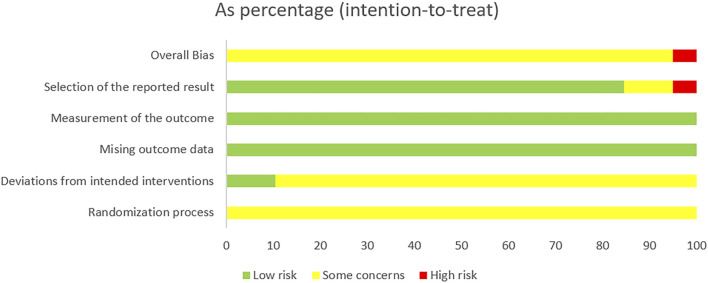
Risk of bias graph.

A total of 39 papers were included in this study, in which seventeen studies (Bai et al., 2022; Chen et al., 2019; Fang et al., 2018; Fu, 2021; Fu et al., 2020; Gong et al., 2021; Lin et al., 2023; Meng, 2018; 2019; Qin et al., 2017; Ren et al., 2023; Shen et al., 2021; Wang B. et al., 2024; Wang, 2022; Wang and Long, 2022; Wang et al., 2019; Zhao D. H. et al., 2021) used the random number table method, 22 studies (Chen et al., 2022; Chu, 2021; Feng et al., 2005; Jiang et al., 2024; Li, 2021; Li and Tang, 2009; Liang et al., 2019; Liu et al., 2003; Liu et al., 2018; Lv et al., 2014; Ma and Xu, 2006; Peng, 2011; Qi et al., 2023; Qin et al., 2023; Sun et al., 2022; Sun, 2021; Wang, 2015; Wei, 2018; Wu et al., 2019; Xie et al., 2019; Zhang, 2022; Zhang et al., 2013) only mentioned randomization without detailing the randomization scheme. No study reported the use of opaque envelopes to conceal the randomization program. Only two studies (Chen et al., 2019; Xie et al., 2019) reported the blinding of participants and researchers; two studies (Liu et al., 2003; Peng, 2011) reported the blinding of participants; seven studies (Feng et al., 2005; Liu et al., 2003; Lv et al., 2014; Ma and Xu, 2006; Peng, 2011; Wang, 2015; Zhang et al., 2013) with small sample sizes. The quality assessment of the included RCTs is shown in Figures 3, 4.

### 3.5 Meta-analysis

#### 3.5.1 Primary outcomes

##### 3.5.1.1 Index of microcirculatory resistance (IMR)

**FIGURE 5:**
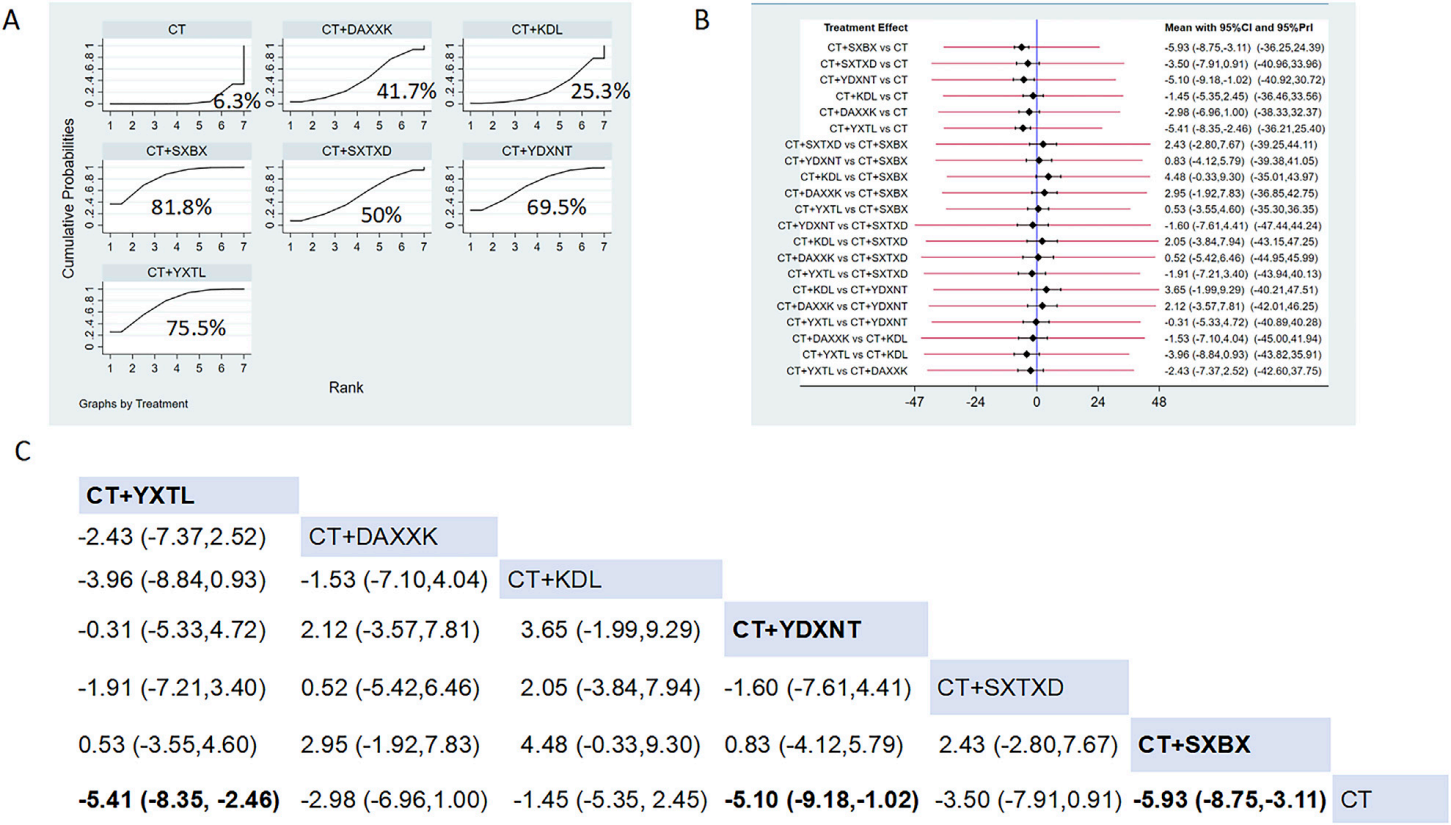
Network meta analysis of IMR in CMD treated with CCPPs. **(A)** SUCRA plot of IMR. A larger SUCRA value indicates a better rank of treatment. **(B)** Forest plot of IMR. The mean difference (MD) is considered statistically significant when the entire 95 % confidence interval does not contain “0”. **(C)** leaque table of IMR. When the entire 95 % confidence interval does not contain “0”, MD is considered statistically significant, which is bolded.

Eight studies reported the effects of nine CCPPs on IMR. Compared with the control group, SXBX [MD = −5.93, 95% CI (−8.75, −3.11)], YXTL [MD = −5.41, 95% CI (−8.35, −2.46)], and YDXNT [MD = −5.10, 95% CI (−9.18, −1.02)] significantly reduced the IMR. However, there was no statistically significant improvement in IMR with SXTXD [MD = −3.50, 95% CI (−7.91, 0.91)], KDL [MD = −1.45, 95% CI (−5.35, 2.45)], and DAXXK [MD = −2.98, 95% CI (−6.96, 1.00)] (Figures 5B,C). According to SUCRA, SXBX may be the most effective intervention to improve IMR (SUCRA = 81.8%), followed by YXTL (SUCRA = 75.5%) and YDXNT (SUCRA = 69.5%) (Figure 5A).

##### 3.5.1.2 Coronary flow reserve (CFR)

**FIGURE 6:**
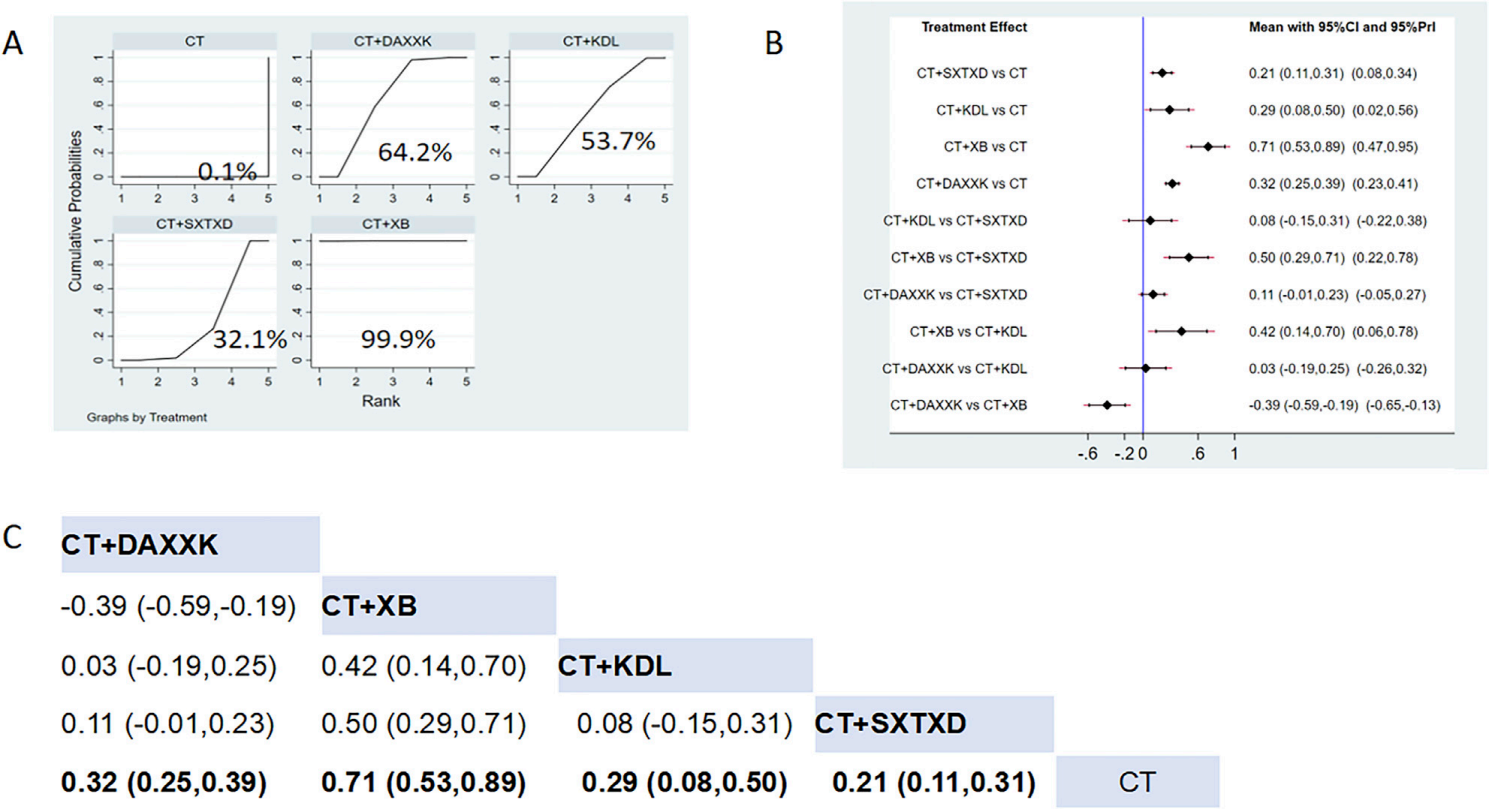
Network meta analysis of CFR in CMD treated with CCPPs. **(A)** SUCRA plot of CFR. A larger SUCRA value indicates a better rank of treatment. **(B)** Forest plot of CFR. The mean difference (MD) is considered statistically significant when the entire 95 % confidence interval does not contain “0”. **(C)** Leaque table of CFR. When the entire 95 % confidence interval does not contain “0”, MD is considered statistically significant, which is bolded.

Five studies reported the effects of nine CCPPs on CFR. Compared with the control group, SXTXD [MD = 0.21, 95% CI (0.11, 0.31)], KDL [MD = 0.29, 95% CI (0.08, 0.50)], XB [MD = 0.71, 95% CI (0.53, 0.89)], and DAXXK [MD = 0.32, 95% CI (0.25, 0.39)] significantly improved the CFR (Figures 6B,C). According to SUCRA, XB may be the most effective intervention to improve CFR (SUCRA = 99.9%), followed by DAXXK (SUCRA = 64.2%), KDL (SUCRA = 53.7%), and SXTXD (SUCRA = 32.1%) (Figure 6A).

#### 3.5.2 Secondary outcomes

##### 3.5.2.1 Angina attack frequency

**FIGURE 7:**
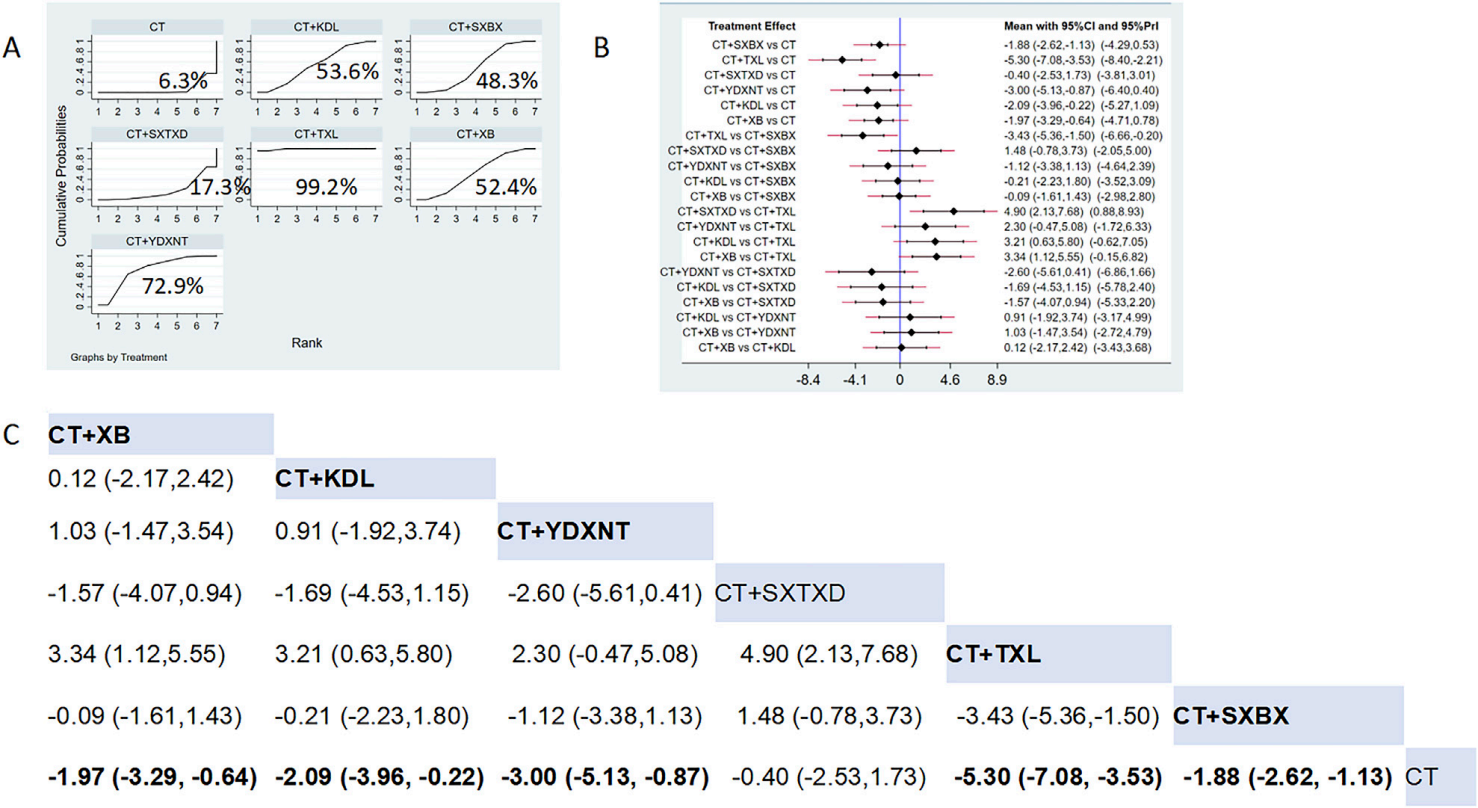
Network meta analysis of Angina attack frequency in CMD treated with CCPPs. **(A)** SUCRA plot of Angina attack frequency. A larger SUCRA value indicates a better rank of treatment. **(B)** Forest plot of Angina attack frequency. The mean difference (MD) is considered statistically significant when the entire 95 % confidence interval does not contain “0”. **(C)** Leaque table of Angina attack frequency. When the entire 95 % confidence interval does not contain “0”, MD is considered statistically significant, which is bolded.

Fourteen studies reported the effects of nine CCPPs on angina attack frequency. Compared with the control group, TXL [MD = −5.30, 95% CI (−7.08, −3.53)], SXBX [MD = −1.88, 95% CI (−2.62, −1.13)], YDXNT [MD = −3.00, 95% CI (−5.13, −0.87)], KDL [MD = −2.09, 95% CI (−3.96, −0.22)], XB [MD = −1.97, 95% CI (−3.29, −0.64)] significantly reduced the angina attack frequency (Figures 7B,C). According to SUCRA, TXL may be the most effective intervention (SUCRA = 99.2%), followed by YDXNT (SUCRA = 72.9%), KDL (SUCRA = 53.6%), XB (SUCRA = 52.4%), and SXBX (SUCRA = 48.3%) (Figure 7A).

##### 3.5.2.2 Hypersensitive C-reactive protein (hs-CRP)

**FIGURE 8:**
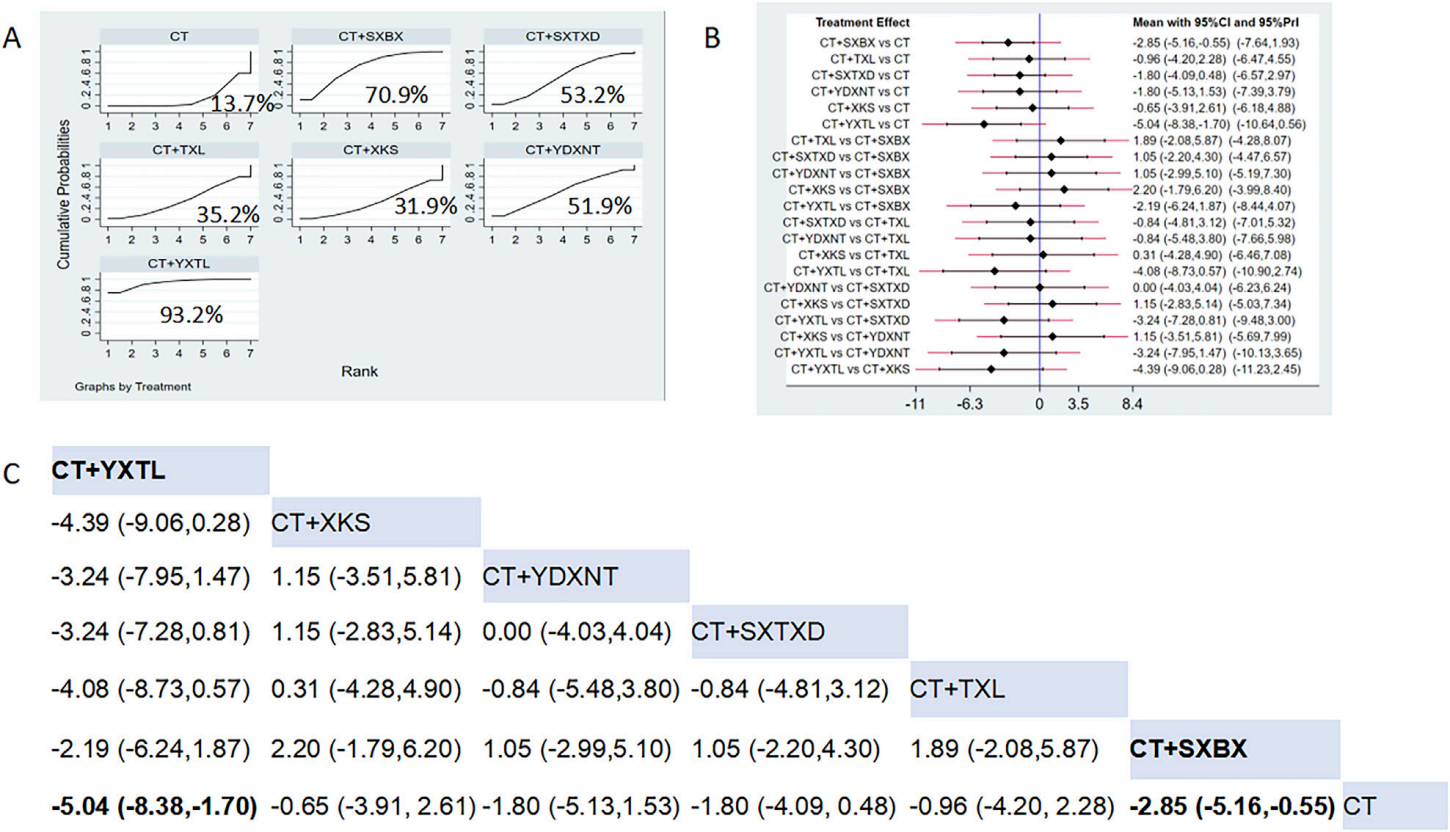
Network meta analysis of hs-CRP in CMD treated with CCPPs. **(A)** SUCRA plot of hs-CRP. A larger SUCRA value indicates a better rank of treatment. **(B)** Forest plot of hs-CRP. The mean difference (MD) is considered statistically significant when the entire 95 % confidence interval does not contain “0”. **(C)** Leaque table of hs-CRP. When the entire 95 % confidence interval does not contain “0”, MD is considered statistically significant, which is bolded.

Eight studies reported the effects of nine CCPPs on hs-CRP. Compared with the control group, YXTL [MD = -5.04, 95% CI (−8.38, −1.7)] and SXBX [MD = -2.85, 95% CI (−5.16, −0.55)] significantly reduced the hs-CRP (Figures 8B,C). According to SUCRA, YXTL may be the most effective intervention to reduce the hs-CRP (SUCRA = 93.2%), followed by SXBX (SUCRA = 70.9%), SXTXD (SUCRA = 53.2%), YDXNT (SUCRA = 51.9%), TXL (SUCRA = 35.2%), and XKS (SUCRA = 31.9%) (Figure 8A).

##### 3.5.2.3 Endothelin-1 (ET-1)

**FIGURE 9:**
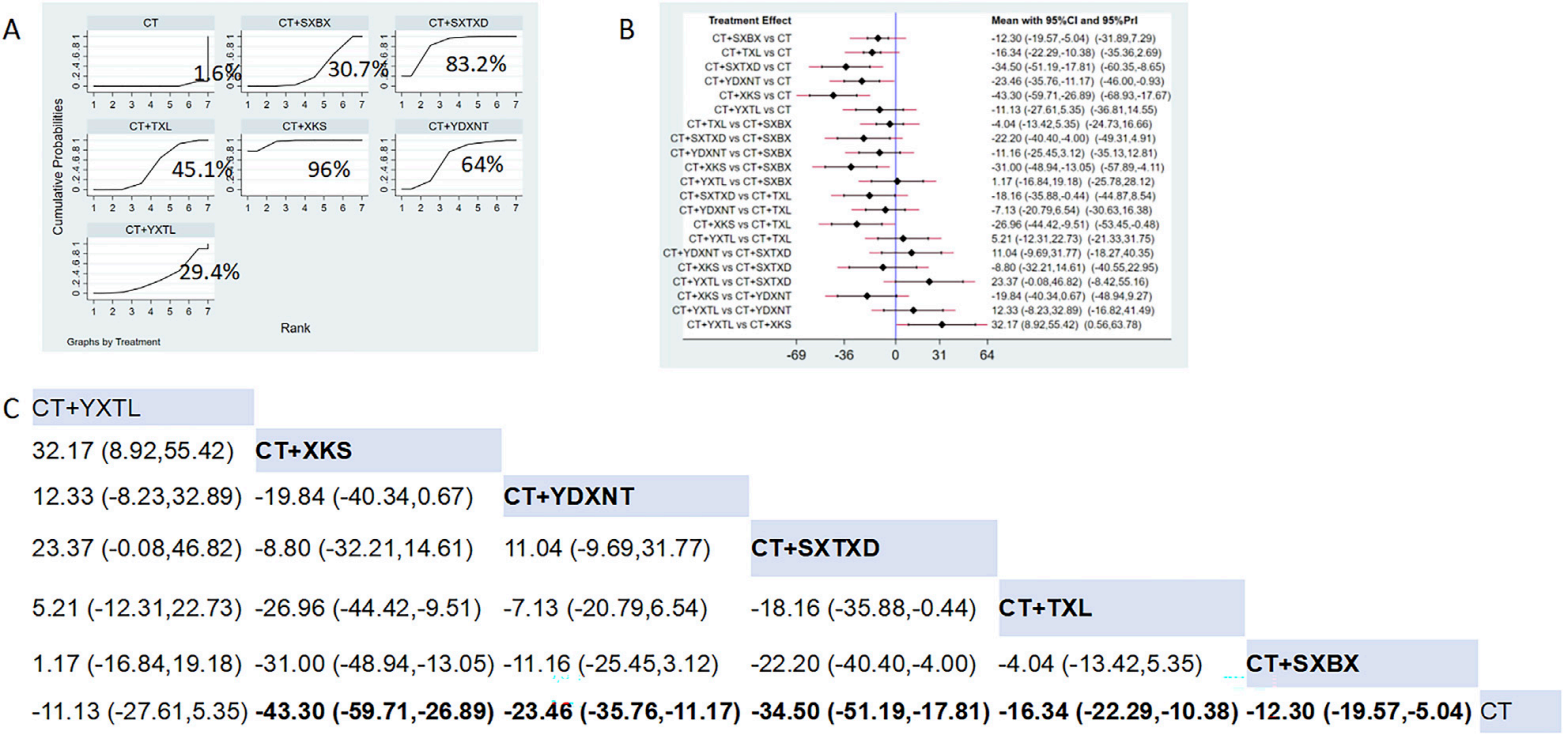
Network meta analysis of ET-1 in CMD treated with CCPPs. **(A)** SUCRA plot of ET-1. A larger SUCRA value indicates a better rank of treatment. **(B)** Forest plot of ET-1. The mean difference (MD) is considered statistically significant when the entire 95 % confidence interval does not contain “0”. **(C)** Leaque table of ET-1. When the entire 95 % confidence interval does not contain “0”, MD is considered statistically significant, which is bolded.

Eighteen studies reported the effects of nine CCPPs on ET-1. Compared with the control group, XKS [MD = −43.3, 95% CI (−59.71, −26.89)], SXTXD [MD = −34.5, 95% CI (−51.19, −17.81)], YDXNT [MD = −23.46, 95% CI (−35.76, −11.17)], TXL [MD = −16.34, 95% CI (−22.29, −10.38)], and SXBX [MD = −12.3, 95% CI (−19.57, −5.04)] significantly reduced the ET-1 (Figures 9B,C). According to SUCRA, XKS may be the most effective intervention to reduce the ET-1 (SUCRA = 96%), followed by SXTXD (SUCRA = 83.2%), YDXNT (SUCRA = 64%), TXL (SUCRA = 45.1%), XSBX (SUCRA = 30.7%), and YXTL (SUCRA = 29.4%) (Figure 9A).

##### 3.5.2.4 Nitric oxide (NO)

**FIGURE 10:**
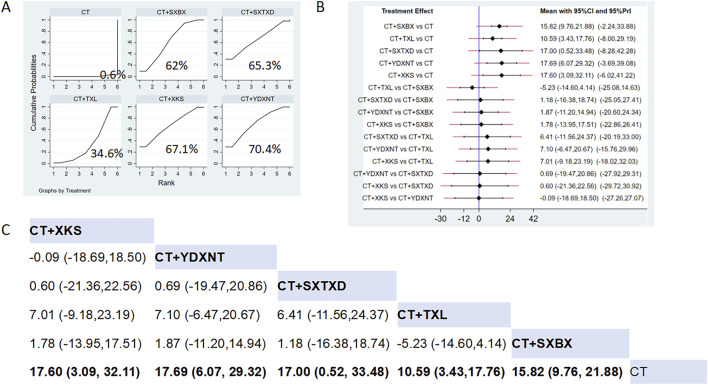
Network meta analysis of NO in CMD treated with CCPPs. **(A)** SUCRA plot of NO. A larger SUCRA value indicates a better rank of treatment. **(B)** Forest plot of NO. The mean difference (MD) is considered statistically significant when the entire 95 % confidence interval does not contain “0”. **(C)** Leaque table of NO. When the entire 95 % confidence interval does not contain “0”, MD is considered statistically significant, which is bolded.

Fifteen studies reported the effects of nine CCPPs on NO. Compared with the control group, YDXNT [MD = 17.69, 95% CI (6.07, 29.32)], XKS [MD = 17.6, 95% CI (3.09, 32.11)], SXTXD [MD = 17.00, 95% CI (0.52, 33.48)], SXBX [MD = 15.82, 95% CI (9.76, 21.88)], and TXL [MD = 10.59, 95% CI (3.43, 17.76)] significantly improved the NO (Figures 10B,C). According to SUCRA, YDXNT may be the most effective intervention to improve the NO (SUCRA = 70.4%), followed by XKS (SUCRA = 67.1%), SXTXD (SUCRA = 65.3%), SXBX (SUCRA = 62%), and TXL (SUCRA = 34.6%) (Figure 10A).

##### 3.5.2.5 Low-density lipoprotein cholesterol (LDL-C)

**FIGURE 11:**
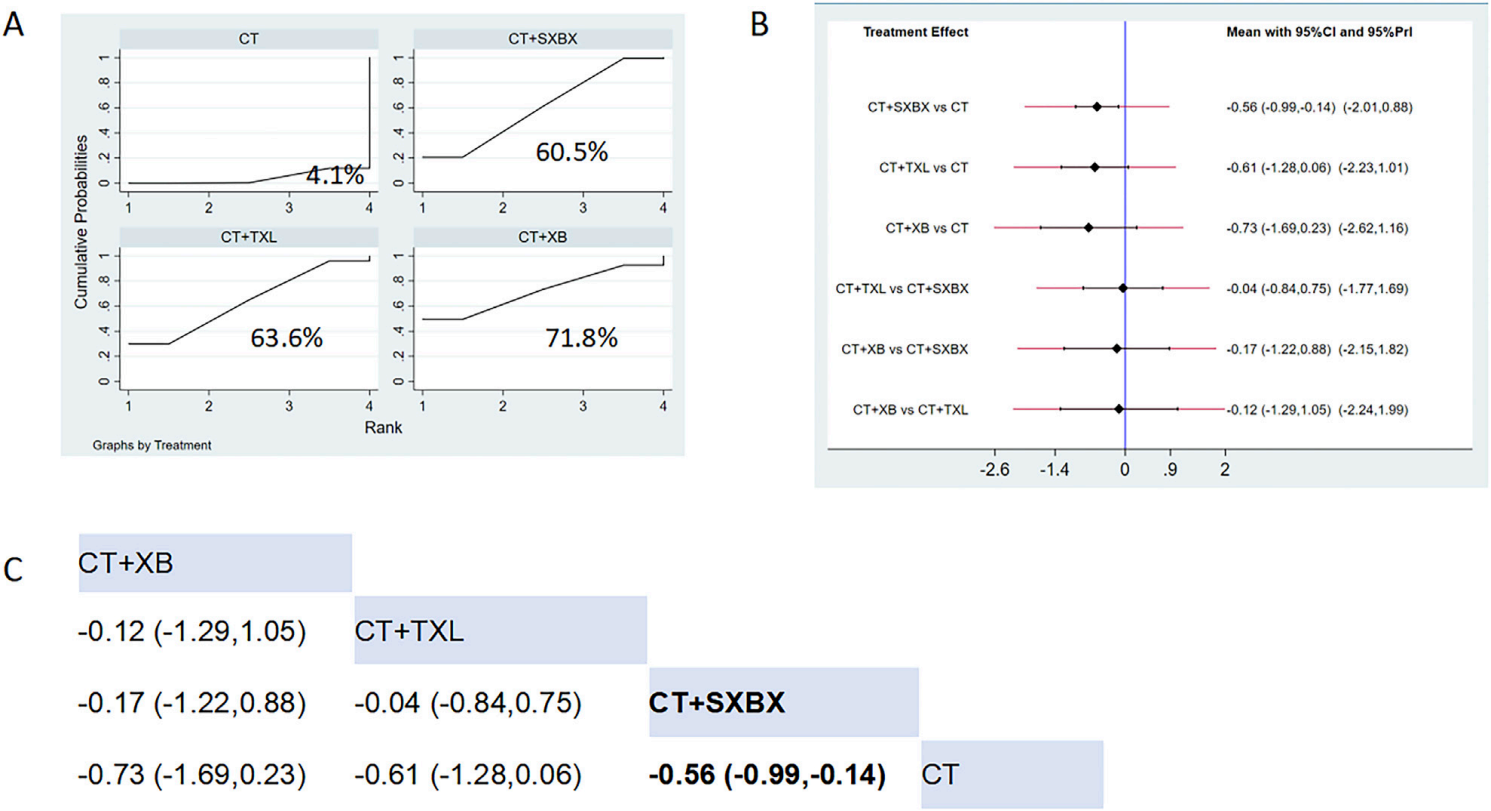
Network meta analysis of LDL-C in CMD treated with CCPPs. **(A)** SUCRA plot of LDL-C. A larger SUCRA value indicates a better rank of treatment. **(B)** Forest plot of LDL-C. The mean difference (MD) is considered statistically significant when the entire 95 % confidence interval does not contain “0”. **(C)** Leaque table of LDL-C. When the entire 95 % confidence interval does not contain “0”, MD is considered statistically significant, which is bolded.

Eight studies reported the effects of nine CCPPs on LDL-C. Compared with the control group, SXBX (MD = −0.56, 95% CI [-0.99, −0.14]) significantly reduced the LDL-C (Figures 11B,C). According to SUCRA, SXBX may be the most effective intervention to reduce the LDL-C (SUCRA = 71.8%) (Figure 11A).

### 3.6 Inconsistency, heterogeneity, meta-regression, and sensitivity analysis

As this network meta-analysis did not form a closed loop, node splitting could not be employed for inconsistency testing. First, we conducted a global consistency test. The results revealed that only the p-values for angina attack frequency, ET-1, and NO were below 0.05, indicating significant inconsistency, which may stem from diagnostic heterogeneity, dosing, follow-up length, and study quality. Secondly, we employed NMA within a frequency-based framework to fit a consistency model. Restricted maximum likelihood (REML) was used to estimate the global heterogeneity variance (τ^2^). Results indicated significant heterogeneity among the included studies. We conducted further meta-regression to identify sources of heterogeneity. Six characteristics were selected, including duration of intervention, CMD diagnosis methods, sample size, gender ratio, year of publication, and risk of bias. However, the regression analyses revealed no significant influence from these covariates, indicating that these characteristics were not sources of heterogeneity between studies. Subsequent sensitivity analyses demonstrated the stability of the results. Finally, we conducted sensitivity analyses excluding high-risk studies and non-validated CMD studies, further demonstrating the robustness of our findings (Supplementary Appendixs S9, S10)

### 3.7 Safety evaluation

**FIGURE 12:**
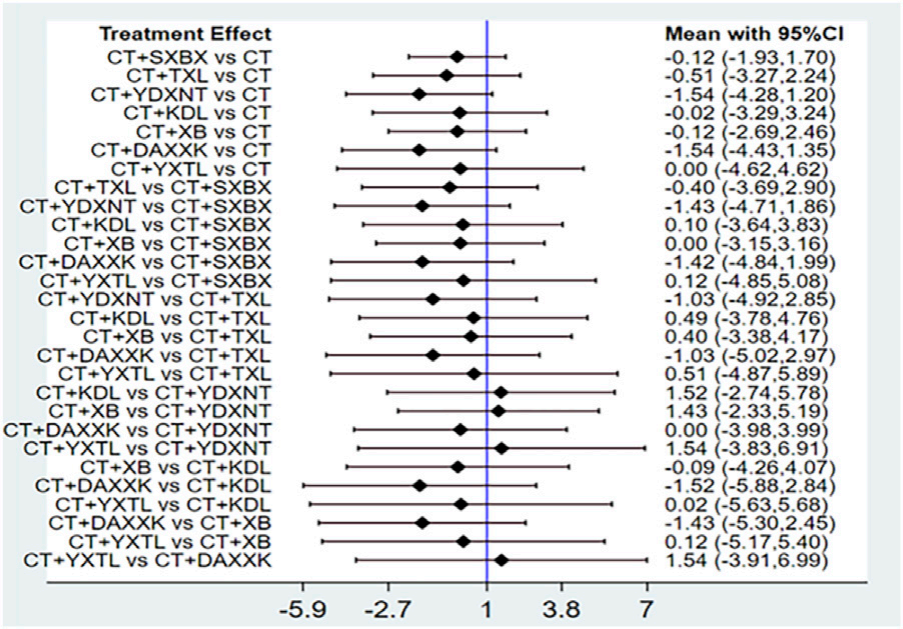
Network meta-analysis of ADRs.

A total of 12 studies reported adverse drug reactions (ADRs), with six studies indicating no ADRs occurred in either the intervention group or the control group. Two studies reported ADRs to SXBX, one study reported ADRs to TXL, one to YDXNT, one to XB, and one to DAXXK. All ADRs were mild, and no study reported withdrawal due to ADRs. The results of the forest plot revealed that there were no significant differences in the risk of adverse drug reactions across various CCPPs (Figure 12). Detailed information is provided in Supplementary Appendix S10.

### 3.8 GRADE assessment

The assessment of the level of evidence for inclusion of the outcomes was summarised using the GRADE methodology as shown in Table 3.

**TABLE 3:** GRADE assessment for the outcomes.

Outcome	Number	Study design	Risk of bias	Inconsistency	Indirectness	Imprecision	Other considerations	Certainty of evidence
CFR	5	RCT	Serious	Not serious	Not serious	Serious	None	⨁⨁○○ Low
IMR	8	RCT	Serious	Not serious	Not serious	Serious	None	⨁⨁○○ Low
Angina attack frequency	14	RCT	Serious	Serious	Not serious	Not serious	None	⨁⨁○○ Low
hs-CRP	8	RCT	Serious	Not Serious	Not serious	Serious	None	⨁⨁○○ Low
ET-1	18	RCT	Serious	Serious	Not serious	Not serious	None	⨁⨁○○ Low
NO	15	RCT	Serious	Serious	Not serious	Not serious	None	⨁⨁○○ Low
LDL-C	8	RCT	Serious	Not Serious	Not serious	Serious	None	⨁⨁○○ Low

### 3.9 Publication bias

**FIGURE 13:**
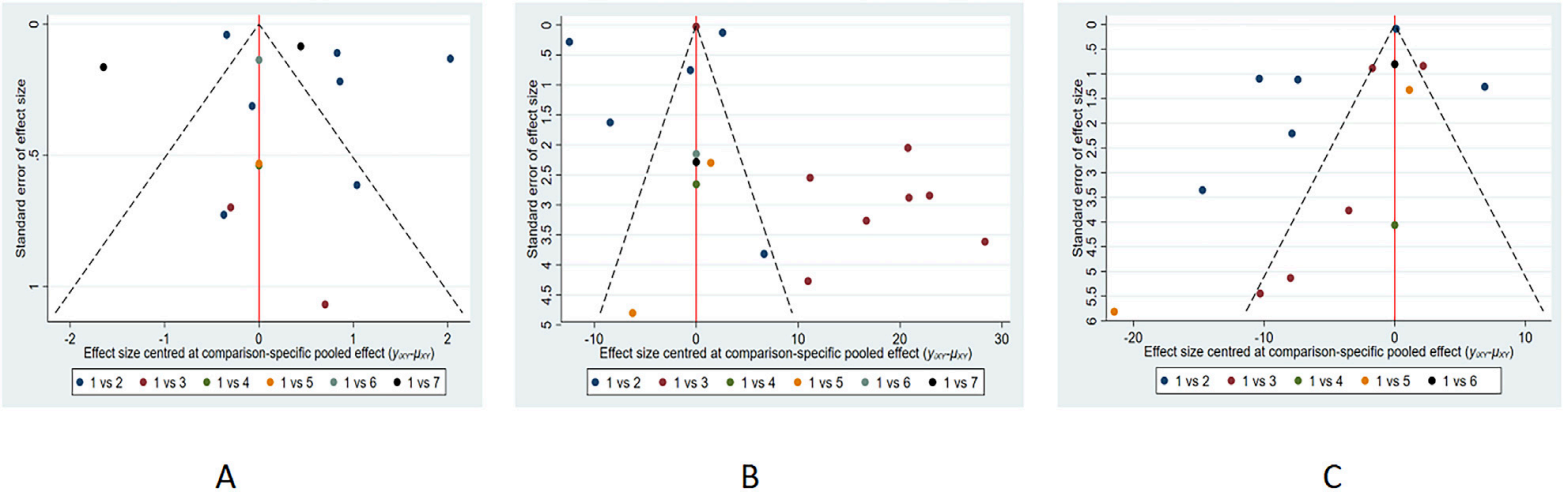
The funnel plot of **(A)** Angina attack frequency; **(B)** ET-1; **(C)** NO.

We assessed publication bias for indicators that included more than ten papers in the study, and the funnel plot results showed that angina attack frequency was roughly symmetrical (Figure 13). The ET-1 and NO funnel plots exhibited poor symmetry, and subsequent Egger’s tests revealed no significant evidence of publication bias (P-values of 0.145, 0.088, and 0.179, respectively). This inconsistency may stem from a potential small-sample effect, or may indicate that funnel plot asymmetry could be attributable to factors beyond publication bias (such as heterogeneity between studies). Nevertheless, we should exercise caution in interpreting the results, as the presence of publication bias cannot be entirely ruled out. It is worth noting that the included small-sample studies generally exhibited low methodological quality (such as more deficiencies in allocation concealment and blinding procedures), and the overestimation of effect sizes may partly stem from this. We have therefore interpreted these findings with caution. Although the possibility of publication bias cannot be ruled out, it is not the sole explanation for this result. It is undeniable that this bias is likely to have substantially impacted our SUCRA ranking results, which rely heavily on unbiased effect estimates. Given the current risk of potential bias, we should emphasize direct comparisons of point estimates and confidence intervals for clinical decision-making, rather than over-relying on specific ranking orders. Therefore, although XKS and YDXNT ranked highest in ET-1 and NO, respectively, further large-scale, high-quality studies are required to validate the relative efficacy of these interventions and provide more reliable evidence for ranking.

For other outcomes with fewer than ten included studies (CFR, IMR, hs-CRP, and LDL-C), formal statistical tests for small-study effects (e.g., Egger’s test) are underpowered. Therefore, the assessment of publication bias for these outcomes relies solely on qualitative interpretation of the funnel plots, which should be considered tentative. More primary studies are needed to allow for robust evaluation of publication bias for these endpoints.

## 4 Discussion

In recent years, there has been a rapid increase in the number of international consensus documents on CMD, and the understanding of CMD has changed (Knuuti et al., 2020; Kunadian et al., 2020; Ong et al., 2018; Padro et al., 2020; Tamis-Holland et al., 2019). In 2023, the Chinese Medical Association issued a Chinese expert consensus on the diagnosis and treatment of coronary microvascular disease (Chen et al., 2023), which classified CMD into four main types and nine subtypes and summarised the diagnostic criteria for different types of CMD. The 2024 ESC Guidelines for the Management of Chronic Coronary Syndromes recommend that patients with recurrent or refractory angina and suspected angina with non-occlusive coronary Arteries (ANOCA)/Ischemia with non-occlusive coronary Arteries (INOCA) undergo invasive coronary functional testing (Class I, Level B) to define underlying endotypes and guide targeted treatment (Vrints et al., 2024). For symptomatic ANOCA/INOCA, the same guidelines advocate a mechanism-guided pharmacologic approach, tailored to the results of coronary functional testing, to optimise symptom control and quality of life. CFR is the coronary or myocardial blood flow ratio during maximal coronary dilatation to the corresponding index at rest. Studies have shown that MACE is higher in patients with CFR <1.6 at 1-year follow-up. CFR is an important predictor of MI and heart failure risk (Taqueti et al., 2015) and an overall indicator of the reserve function of the entire coronary system. IMR is an index of myocardial microcirculatory function in coronary arteries at maximal microcirculatory dilatation measured by a pressure/temperature guidewire (Ng et al., 2012). IMR and CFR are common invasive means to detect microcirculatory function. Therefore, we chose CFR and IMR as the primary outcomes to compare the protective effect of CCPPs on the reserve function of the entire coronary system. The main symptom of CMD is angina pectoris, so we chose the frequency of angina attacks to indicate the effect of various CCPPs on the clinical symptoms of CMD patients. The pathological mechanisms of CMD have not been fully elucidated. However, oxidative stress and inflammatory responses caused by excessive production and accumulation of cellular reactive oxygen species are considered to be the key pathogenic mechanisms driving the development of CMD (Masi et al., 2021), and dyslipidemia also plays an important role in the occurrence and development of CMD (Padro et al., 2020). Therefore, we chose ET-1, NO, hs-CRP, and LDL-C as the indices reflecting the effects of various CTMs on endothelial function, inflammation, and lipids.

### 4.1 Summary of findings

A total of 39 RCTs involving 3,240 patients were included in the study. NMA results showed that the efficacy of CT combined with CCPPs was significantly better than CT alone. SXBX had the highest probability of being the best treatment on account of the reduction of IMR [MD = −5.93, 95% CI (−8.75, −3.11)] and LDL [MD = −0.56, 95% CI (−0.99, −0.14)]; XB showed better efficacy in CFR [MD = 0.71, 95% CI (0.53, 0.89)]; TXL showed better efficacy in angina attack frequency [MD = −5.30, 95% CI (−7.08, −3.53)]; YXTL showed better efficacy in hs-CRP [MD = −5.04, 95% CI (−8.38, −1.7)]; XKS showed better efficacy in ET-1 [MD = −43.3, 95% CI (−59.71, −26.89)]; YDXNT showed better efficacy in NO [MD = 17.69, 95% CI (6.07, 29.32)]. However, this finding must be interpreted with extreme caution, as the GRADE assessment indicates that the quality of evidence for all comparisons is low. This implies that our confidence in the accurate estimate of the effect size is limited, and future research is likely to alter or even reverse the current ranking and conclusions.

### 4.2 Ingredients of CCPPs and frequently used herbs

CMD is classified in TCM under “Xiong Bi” and “Xin Tong” (angina pectoris). Blood-activating and Qi-promoting CCPPs have been reported to improve coronary microcirculatory and vascular endothelial functions and alleviate pain. Our NMA is the first to compare various CCPPs in CMD systematically. The study highlighted differences in efficacy, but all shared the core TCM principle of “Blood Activation and Qi Promotion.” Furthermore, analysis of the composition of each CCPP revealed that the most frequently used herbs were Ginseng, Salvia miltiorrhiza, Panax notoginseng, Artificial musk, and Borneol. They benefit qi, improve blood circulation, and relieve pain. These herbs may offer potential therapeutic benefits for CMD. However, the exact mechanisms behind their effects require further investigation through modern pharmacological research.

### 4.3 Possible mechanism of herbal benefits for CMD

Several CCPPs demonstrated significant efficacy in our NMA for CMD. Their benefits appear to stem from both the active herbal components they contain and the multi-target mechanisms these formulations employ. Below, we first discuss the major CCPPs and their pharmacological effects and then provide an overview of commonly used single herbal compounds.

### 4.4 Representative CCPPs

SXBX is an aromatic and warming CCPP that benefits Qi and strengthens the heart. It was the most effective CCPP for decreasing IMR, which may be due to its effects in reducing lipid levels, plaque formation, and endothelial damage (Li D. et al., 2024), anti-inflammation, anti-atherosclerosis (Lu et al., 2019), and protection of endothelial function (Ning et al., 2011). Studies have shown (Wei et al., 2023) that it can promote angiogenesis via the GDF15-TRPV4 signaling pathway. It inhibits pyroptosis and improves I/R injury by promoting autophagosome generation and accelerating autophagic flux (Yu et al., 2022). Comprehensive metabolomics studies have shown it protects cardiac function by regulating amino acid, lipid, and energy metabolisms (Wu et al., 2020).

XB was the most effective CCPP in increasing CFR, possibly due to its improved energy metabolism, suppressed apoptosis, suppressed excessive autophagy, and endoplasmic reticulum (ER) stress effects. It has been shown to inhibit SGLT1 protein expression while upregulating the phosphorylation level of AMPK, promoting nuclear translocation of PPARα and enhancing its transcriptional activity, ultimately improving fatty acid energy metabolism in the heart (Pan et al., 2024). It also promotes mitochondrial homeostasis by inhibiting heme synthesis to increase succinyl-CoA (Chen et al., 2025). In addition, XB inhibits excessive autophagy by decreasing Beclin-1 and LC3II and increasing p62. It also inhibits ER stress by decreasing BIP expression and apoptosis by increasing Bcl2/Bax and decreasing caspase3 (Yang et al., 2022). DAXXK is second only to XB in increasing CFR. The main ingredient of DAXXK is total steroidal saponin, which is an Rhizome extract of Dioscorea nipponica Makino, and has been shown to reduce TC and TG levels, anti-inflammatory, anti-oxidative stress, and anti-atherosclerotic effects (Zhang et al., 2022).

TXL ranked highest for reducing angina attack frequency. It has the function of invigorating qi and promoting blood circulation, which can enhance myocardial contractility, inhibit platelet aggregation, and regulate the level of blood lipids (Chen et al., 2024). Studies have shown it could alleviate no-reflow by suppressing the interactions by modulating various leukocyte subtypes and inhibiting the expression of multiple inflammatory mediators (Liu S. et al., 2023). TXL also inhibited endothelial cell pyroptosis via the reactive oxygen species/nucleotide-binding oligomerization domain-like receptor family pyrin domain-containing 3/Caspase-1/GSDMD signalling pathway (Gu et al., 2023). YXTL ranked highest for decreasing hs-CRP levels. It has the effects of improving coronary microcirculation, anti-inflammation, and anti-platelet aggregation (Meng, 2020). KDL is a processed tablet made from Corydalis Rhizoma, which has anti-myocardial ischaemia, anti-thrombotic, and anti-arrhythmic effects (Sun et al., 2009).

XKS ranked highest for decreasing ET-1 levels, It can also elevate the nitric oxide content, improve the vascular endothelial function (Liu et al., 2022). Studies (Liu et al., 2016) have shown it protects cardiac function by inhibiting the myocardium Ca (2+) overloading and metabolic alterations. It also promotes angiogenesis through multiple signaling pathways, including metabolic pathways, the NOD-like receptor signaling pathway, the VEGF signaling pathway, the PPAR signaling pathway, and the PI3K/Akt signaling pathway (Liu Q. et al., 2023). SXTXD is second only to XKS in reducing ET-1. It can regulate the cellular autophagy process, promote smooth muscle cell proliferation and differentiation, exert anti-inflammatory effects, and optimize lipid metabolism (Chang et al., 2022).

YDXNT ranked highest for increasing NO levels. Studies have shown it has the effect of repairing damaged endothelial cells, reducing the release of endothelin, dilating blood vessels, and improving coronary microcirculation (Yang et al., 2023); it also can exert anti-inflammatory effects by decreasing IL-1β, IL-8, and IL-18 via the TLR4 pathway (Wang et al., 2014). In addition, it relieves atherosclerosis through regulating lipids, reducing lipid particle deposition in the endothelial layer of the artery, enhancing antioxidant power, and repressing inflammatory activity by inhibiting the nuclear factor-kappa B signal pathway (Cheng et al., 2015).

Overall, these commonly used herbs and their preparations appear to address key pathophysiological mechanisms of CMD, including oxidative stress, inflammatory responses, atherosclerosis, vascular endothelial dysfunction, and abnormal energy metabolism. By combining multiple active ingredients, CCPPs may offer synergistic effects; however, further well-designed studies are needed to determine optimal dosages, assess long-term safety, and elucidate their clinical utility.

### 4.5 Key active herbal components

**FIGURE 14:**
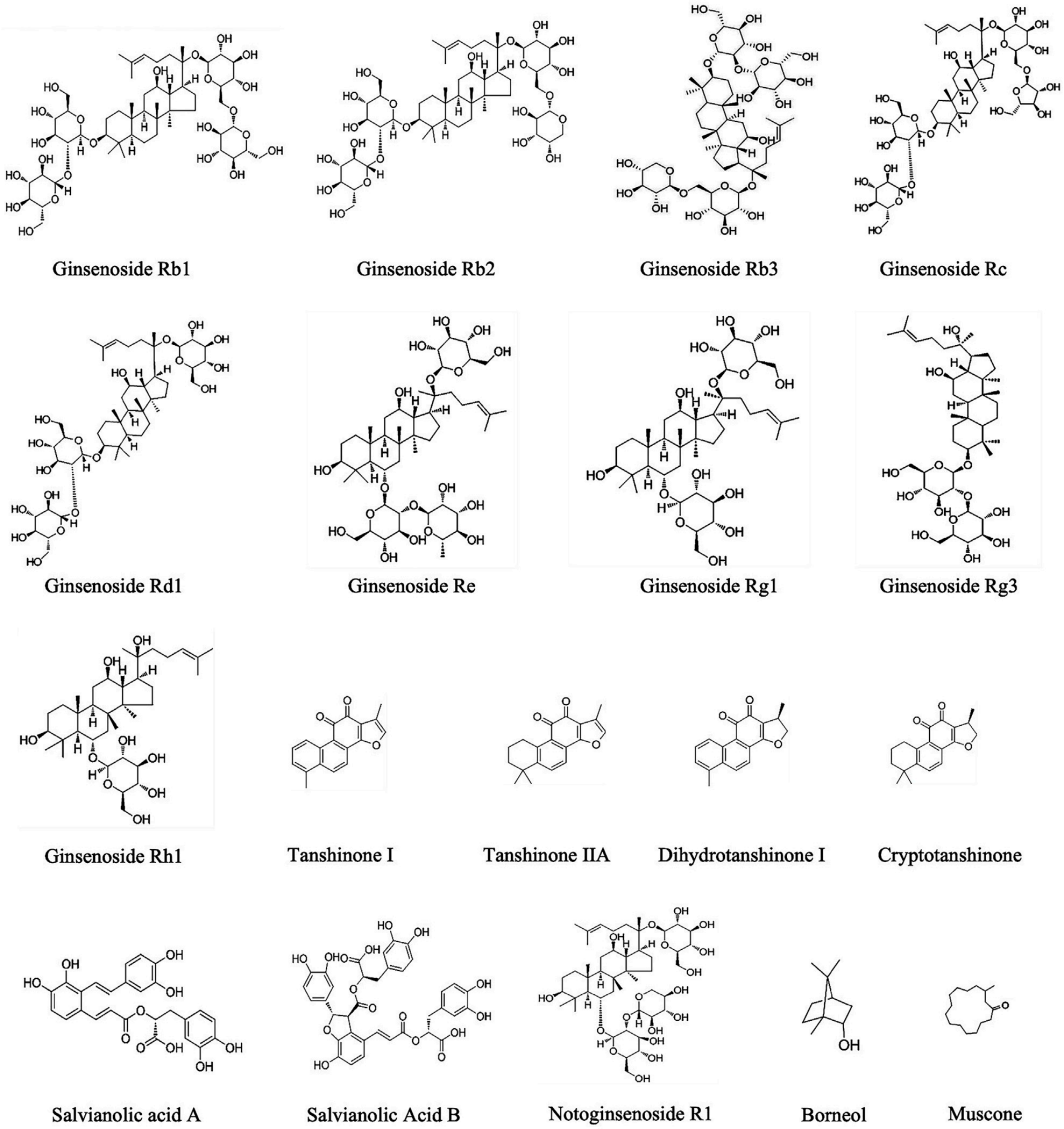
The structural formula of the main active components.

Our study’s most frequently used herbs were Panax ginseng, Salvia miltiorrhiza, Panax notoginseng, Moschus, and Borneolum. A total of 45 experimental studies were identified to investigate the effects and mechanisms of the main active components of single-flavored Chinese medicine, which were frequently used in CMD. Table 4 lists the Mechanisms of the main active components of single-flavored Chinese Medicine on CMD. The structural formula of the main active components are showed in Figure 14. The possible mechanisms of them are summarized as follows:

**TABLE 4:** Mechanisms of the main active components of single-flavored Chinese Medicine on CMD.

Metabolites	Source	Possible mechanisms	References
Ginsenoside Rb_1_	*Panax ginseng* C. A. Mey	1. Anti-Atherosclerosis (induction of macrophage autophagy via Promotion of AMPK Phosphorylation) 2. Protection of vascular endothelium (via p38/JNK/eNOS/NO pathway) 3. Anti-inflammation (by inhibiting MAPK signaling and MEK1/2 activation; suppressing STING-mediated macrophage activation) 4. Promote angiogenesis (increase the expression of VEGF)	1. (Qiao et al., 2017) 2. (Chen et al., 2010) 3. (Wang et al., 2021a) 4. (Liu et al., 2008)
Ginsenoside Rb_2_	*Panax ginseng* C. A. Mey	1. Anti-apoptosis (Nrf2/HO-1 pathway)	1. (Li and Zhang, 2022)
Ginsenoside Rb3	*Panax ginseng* C. A. Mey	1. Anti-oxidant stress (decrease MDA and increase SOD)	1. (Liu et al., 2014)
Ginsenoside Rc	*Panax ginseng* C. A. Mey	1. Anti-Atherosclerosis (regulating gut microbiota and fecal metabolites) 2. Anti-oxidant stress (decrease MDA and increase GSH via Nrf2/HO-1Signaling Pathway)	1. (Xie et al., 2022) 2. (Shi et al., 2022)
Ginsenoside Rd1	*Panax ginseng* C. A. Mey	1. Anti-inflammation (PI3K/Akt Signaling Pathway)	1. (Wang et al., 2024d)
Ginsenoside Re	*Panax ginseng* C. A. Mey	1. Improving energy metabolism (regulating mitochondrial biogenesis through Nrf2/HO-1/PGC-1α pathway)2. Inhibition of aberrant proliferation and migration of VSMCs (via the eNOS/NO/cGMP pathway)	1. (Xin et al., 2024) 2. (Gao et al., 2019)
Ginsenoside Rg1	*Panax ginseng* C. A. Mey	1. Inhibition of aberrant proliferation and migration of VSMCs (via the PKC-zeta and p21 pathway) 2. Improving energy metabolism (binds to RhoA and downregulates the activity of RhoA/ROCK signaling pathway) 3. Anti-oxidant stress (reduce intracellular ROS and increase T-SOD, CAT, and GSH)	1. (Ma et al., 2006) 2. (Li et al., 2018) 3. (Zhu et al., 2009)
Ginsenoside Rg3	*Panax ginseng* C. A. Mey	1. Anti-inflammation (repressing NLRP3 inflammasome via SIRT1/NF-κB pathway) 2. Anti-oxidant stress (increase GSH-Px、SOD and CAT, decrease MDA and ROS)	1. (Ren et al., 2021) 2. (Zhao et al., 2021b)
Ginsenoside Rh1	*Panax ginseng* C. A. Mey	1. Improving energy metabolism (upregulates SIRT3/Foxo3a pathway)	1. (Gong et al., 2025)
Tanshinone I	*Salvia miltiorrhiza* Bunge	1. Anti-oxidant stress (Nrf2 Signaling pathway)	1. (Wu et al., 2021)
Tanshinone IIA	*Salvia miltiorrhiza* Bunge	1. Protection of vascular endothelium (via the TRPV4-NO-PKG signaling pathway) 2. Anti-inflammation (decrease IL-6 and TNF-α via TLR4/TAK1/NF-κB pathway) 3. Improving energy metabolism (increases the expression of 14-3-3η and regulates the Akt/Beclin1 pathway) 4. Anti-oxidant stress (reduce ROS and MDA via inhibiting CLIC1 expression and membrane translocation) 5. Promote angiogenesis (increase the expression of VEGF)	1. (Wang et al., 2024c) 2. (Meng et al., 2019) 3. (Wen et al., 2023) 4. (Zhu et al., 2017) 5. (Xu et al., 2009)
Dihydrotanshinone I	*Salvia miltiorrhiza* Bunge	1. Anti-inflammation (decrease TNF-α, IL-1β, and IL-6 via TLR4-MyD88-NF-κB/MAPK pathway) 2. Anti-platelet (suppression of [Ca2+]i mobilization and arachidonic acid liberation)	1. (Yuan et al., 2019) 2. (Park et al., 2008)
Cryptotanshinone	*Salvia miltiorrhiza* Bunge	1. Anti-inflammation (decrease TNF-α,IL-6 via TLR4-MyD88/PI3K/Nrf2 and TLR4-MyD88/NF-κB/MAPK pathways) 2. Anti-platelet (PI3K/AKT signaling pathway)	1. (Li et al., 2020) 2. (Xiao et al., 2025)
Salvianolic acid A	*Salvia miltiorrhiza* Bunge	1. Anti-inflammation (decrease TNF-α and IL-6 via the p38-HO-1 pathway) 2. Anti-Atherosclerosis (metabolic-dependent anti-EndMT pathway and repression of TGF-β/ALK5 signaling) 3. Anti-oxidant stress (reduce LDH, ROS and increase SOD by downregulating miR-204-5p)	1. (Huang et al., 2013) 2. (Gao et al., 2025) 3. (Qiao et al., 2024)
Salvianolic Acid B	*Salvia miltiorrhiza* Bunge	1. Anti-inflammation (decrease IL-1β、IL-6、IL-8 via inhibiting the activation of NF-κB) 2. Anti-Atherosclerosis (promote the expression of tRF-Glu-CTC-014) 3. anti-platelet (directly blocks the thrombin catalytic site)	1. (Xu et al., 2015) 2. (Chang et al., 2024) 3. (Neves et al., 2024)
Notoginsenoside R1	*Panax notoginseng* (Burkill) F. H. Chen	1. Promote angiogenesis (decreased the hypermethylation of microRNA 200a and increase the expression of VEGF; activates the Ang2/Tie2 pathway) 2. Anti-inflammation and calcification primarily (via the NO-TGFβR1-YAP/TAZ signaling pathway) 3. Anti-Atherosclerosis (inhibition of ferroptosis via Keap1/Nrf2 signaling pathway) 4. Protection of vascular endothelium (downregulating the MyD88/TRAF6/NF-κB pathway via upregulating miR-147a) 5. anti-platelet (via AA/COX-1/TXB2 pathway)	1. (Wang et al., 2024b) 2. (Cui et al., 2024) 3. (Zhao et al., 2024) 4. (Li and Huang, 2021) 5. (Wang et al., 2021b)
Borneol	Borneolum	1. Anti-Atherosclerosis (via inhibiting macrophage foam-cell formation −/−) 2. Protection of vascular endothelium (reduce LDH, MDA, GSSG, and increase GSH) 3. Anti-inflammation (decrease IL-1β、IL-6) 4. Promote angiogenesis (HIF-1α/VEGF signaling pathway)	1. (He et al., 2025) 2. (Mao and Cai, 2022) 3. (Wang et al., 2022) 4. (Wang et al., 2022)
Muscone	Moschus	1. Anti-inflammation (via NF-κB/p65 pathway) 2. Anti-oxidant stress (reduce MDA, LDH and increase SOD) 3. Promote angiogenesis (HIF-1α/VEGF signaling pathway)	1. (Li et al., 2024b) 2. (Wu et al., 2011) 3. (Du et al., 2018)

JNK, Jun N-terminal kinase; eNOS, endothelial nitric oxide synthase; NO, nitric oxide; MAPK, Mitogen-Activated Protein Kinase; MEK1/2, Mitogen-activated protein kinase kinases 1 and 2; STING, stimulator of interferon genes; VEGF, vascular endothelial growth factor; Nrf2, Nuclear factor erythroid 2-related factor 2; HO-1, Heme Oxygenase-1; MDA, malonaldehyde; SOD, super oxide dismutase; GSH, glutathione; PI3K, phosphatidylinositol 3-kinase; AKT, Protein Kinase B; PGC-1α, peroxisome proliIerators-activated receptor γ coactivator lalpha; VSMCs, Vascular Smooth Muscle Cell; cGMP, current good manufacture practices; PKC, Protein Kinase C; RhoA, Ras Homolog Family Member A; ROCK, Rho-associated coiled-coil-containing protein kinase; CAT, catalase; NLRP3, NOD-, LRR- and, pyrin domain-containing protein 3; SIRT1, silent information regulator sirtuin 1; NF-κB, nuclear factor kappa-B; TRPV4, Transient Receptor Potential Cation Channel Subfamily V Member 4; TNF-α, Tumor Necrosis Factor-α; TLR4, Toll-like receptor 4; TAK1, Transforming Growth Factor-β-Activated Kinase 1; CLIC1, Chloride Intracellular Channel 1; VEGF, vascular endothelial growth factor; IL-1β, Interleukin-1beta; MyD88, Myeloid differentiation primary response protein 88; EndMT, endothelial-mesenchymal transition; TGF-β, transforming growth factor-β; ALK5, Activin receptor-like kinase 5; LDH, lactate dehydrogenase; ROS, reactive oxygen species; YAP, Yes-associated protein; TAZ, Transcriptional coactivator with PDZ-binding motif; Keap1, Kelch-like ECH-associated protein 1; TRAF6, TNF, receptor associated factor 6; AA, arachidonic acid; COX-1, Cyclooxygenase −1; TXB-2, thromboxane-2; GSSG, glutathione, Oxidized; HIF-1α, hypoxia inducible factor-1.

#### 4.5.1 Anti-atherosclerosis

Structural changes in the coronary microcirculation include remodelling and narrowing of the microvasculature, which ultimately leads to an increase in coronary microcirculatory resistance and a decrease in coronary blood flow. Therefore, anti-atherosclerosis is considered an important step in the prevention of CMD. Studies have shown that Ginsenoside Rb1 and Borneol ameliorated atherosclerosis via inhibiting macrophage foam-cell formation −/− (He et al., 2025; Qiao et al., 2017); Ginsenoside Rc ameliorated atherosclerosis via regulating gut microbiota and faecal metabolites (Xie et al., 2022); Salvianic acid A ameliorates atherosclerosis through metabolic-dependent anti-EndMT pathway and repression of TGF-β/ALK5 signaling (Gao et al., 2025); Salvianic acid B can promote the expression of tRF-Glu-CTC-014 to treat atherosclerosis (Chang et al., 2024); Panax notoginseng saponins (PNS) mitigates atherosclerosis via promoting Nrf2-mediated inhibition of ferroptosis through reducing USP2-mediated Keap1 deubiquitination (Zhao et al., 2024).

#### 4.5.2 Inhibition of aberrant proliferation and migration of VSMCs

Vascular smooth muscle cells (VSMCs) are considered a major component of the vascular wall and regulators responsible for maintaining vascular tension. During ischaemia-reperfusion, activation of MAPK and inflammation-related signalling pathways induces abnormal proliferation and migration of VSMC, the latter being a key event in the development of atherosclerotic lesions, which leads to narrowing of the microvascular lumen. One study reported that GS-Re could inhibit the proliferation of VSMCs by mediating G0/G1 cell cycle arrest via eNOS/NO/cGMP signalling pathway (Gao et al., 2019); another study reported that Ginsenoside Rg1 (Ma et al., 2006) could inhibit the proliferation of VSMCs via the PKC-zeta and p21 pathway.

#### 4.5.3 Protection of the vascular endothelium

Endothelial dysfunction is one of the major mechanisms of CMD, which can be classified as Impaired endothelium-dependent vasodilation or Impaired endothelium-independent vasodilation. The former is mainly caused by stimuli such as cigarette smoking, hypertension, hyperglycaemia, chronic inflammation, and other stimuli induced by vascular endothelial injury, resulting in a decrease in endothelium-mediated diastolic capacity. The latter mainly involves the decreased reactivity of coronary arteries to vasodilating substances. One study reported that Ginsenoside Rb1 could effectively block resistin-induced eNOS downregulation and ROS production (Chen et al., 2010); One study reported that Notoginsenoside R1 could relieve HG-induced endothelial cell injury by downregulating the MyD88/TRAF6/NF-κB pathway via upregulating miR-147a (Li and Huang, 2021); Borneol (Mao and Cai, 2022) could reduce LDH, MDA, and increase GSH, thereby attenuating oxidative stress-induced endothelial damage. One study reported Tanshinone IIA (Wang P. et al., 2024) could induce endothelium-dependent vasodilation via the TRPV4-NO-PKG signaling pathway; another study (Chang et al., 2014) reported Magnesium lithospermate B, an active extract of Salvia miltiorrhiza, could exert anti-vascular spasm through the sGC/cGMP/PKG pathway.

#### 4.5.4 Anti-inflammation

Inflammation and endothelial dysfunction have been shown to be the underlying causes of CMD. Microcirculation is both an important participant in and responsive to the inflammatory response; inflammation can lead to increased vascular permeability and impaired vasomotor function. Ginsenoside Rb1 (Wang S. et al., 2021), Tanshinone II (Meng et al., 2019), dihydrotanshinone I (Yuan et al., 2019), Cryptotanshinone (Li et al., 2020), Salvianolic acid A (Huang et al., 2013), Salvianolic Acid B (Xu et al., 2015), Borneol (Wang et al., 2022), and Muscone (Li L. et al., 2024) were shown to exert anti-inflammatory effects by decreasing interleukin-1beta (IL-1β), IL-6, tumor necrosis factor-alpha (TNF-α), and NF-κB; One study (Wang Y. et al., 2024) reported that Ginsenoside Rd1 exhibits anti-inflammatory effects via PI3K/Akt Signaling Pathway; Ginsenoside Rg3 (Ren et al., 2021) represses NLRP3 inflammasome via SIRT1/NF-κB pathway; Notoginsenoside R1 (Cui et al., 2024) exhibits anti-inflammatory effects via the NO-TGFβR1-YAP/TAZ signaling pathway.

#### 4.5.5 Antioxidant stress

Oxidative stress and inflammatory responses caused by the overproduction and accumulation of reactive oxygen species (ROS) are the key pathogenic mechanisms driving the development of CMD (Masi et al., 2021). The resulting damage to coronary microvascular endothelial cells is a central part of this process (Del Buono et al., 2021). Studies have shown that Ginsenoside Rb3 (Liu et al., 2014), muscone (Wu et al., 2011) could decrease MDA and increase SOD; Ginsenoside Rg1 (Zhu et al., 2009) could reduce intracellular ROS and increase SOD, CAT, and GSH; Three studies reported Ginsenoside Rc (Shi et al., 2022), Ginsenoside Rg3 (Zhao Y. et al., 2021), and Tanshinone I (Wu et al., 2021) Inhibits Oxidative Stress-Induced Cardiomyocyte Injury by Modulating Nrf2/HO-1 Signaling; One study reported Tanshinone IIA (Zhu et al., 2017) Inhibits Oxidative Stress via inhibiting CLIC1 expression and membrane translocation; and One study reported Salvianolic acid A (Qiao et al., 2024) reduce LDH, ROS and increase SOD by downregulating miR-204-5p.

#### 4.5.6 Improving energy metabolism

Ischaemia and hypoxia can impair energy metabolism, causing increased endothelial cell apoptosis, autophagy hyperactivation, and dysfunction. Two studies reported that Ginsenoside Rb1 (Li and Zhang, 2022) and Ginsenoside Re can significantly reduce I/R injury through the Nrf2/HO-1/PGC-1α pathway, thereby increasing the number of mitochondria, improving mitochondrial function, enhancing the ability of cells to resist oxidative stress, and alleviating cell apoptosis (Xin et al., 2024); One study reported that Ginsenoside Rh1 mitigates mitochondrial dysfunction induced by myocardial ischaemia through activating sirtuin 3 (Gong et al., 2025); Tanshinone IIA increases the expression of 14-3-3η and regulates the Akt/Beclin1 pathway, thereby inhibiting excessive autophagy during ischemia and hypoxia, improving mitochondrial energy supply, and ultimately protecting cells from injury (Wen et al., 2023); A study showed that Rg1 binds to RhoA and downregulates the activity of the RhoA signalling pathway to regulate energy metabolism and inhibit myocardial apoptosis (Li et al., 2018).

#### 4.5.7 Antiplatelet activation and aggregation

Microthrombi are one of the mechanisms causing coronary microcirculatory dysfunction. In particular, microthrombi and plaque fragments generated by treatment during percutaneous coronary intervention may lead to distal microvascular occlusion. One study reported that 15,16-Dihydrotanshinone I could exert potent anti-platelet activity by suppressing [Ca2+]i mobilization and arachidonic acid liberation (Park et al., 2008); Cryptotanshinone could effectively inhibit platelet activation in a manner that is independent of the P2Y12 receptor, and the effects appeared to be mediated through intricate signaling pathways, including PI3K-AKT, MAPK, and STAT3 (Xiao et al., 2025); Salvianolic acid B could inhibit thrombosis by directly blocking the catalytic site of thrombin (Neves et al., 2024); One study demonstrated that the combination of PNS and aspirin potentiated the antiplatelet effect of aspirin via AA/COX-1/TXB_2_ pathway in platelets (Wang W. et al., 2021).

#### 4.5.8 Promote angiogenesis

In Coronary microvascular disease, the decreased production of NO by impaired endothelial cells also increases collagen deposition, reduces angiogenesis and collateral development, and promotes the conversion of endothelial cells into mesenchymal cells, leading to microvascular rarefaction (Vancheri et al., 2020). Vascular endothelial growth factor (VEGF) is an important regulator of microvascular neovascularisation, which induces the division of CMECs into newborns, promotes the establishment of collateral circulation, and meets part of the metabolic needs of ischemic cardiomyocytes. One study reported that Ginsenoside Rb1 increased the expression of VEGF (Liu et al., 2008), Three studies reported Tanshinone IIA (Xu et al., 2009), Muscone (Du et al., 2018), and Borneol (Wang et al., 2022) could promote angiogenesis via the HIF-1α/VEGF signaling pathway; Panax notoginseng Saponins could promote angiogenesis via the microRNA 200a Methylation Pathway (Wang J. et al., 2024).

### 4.6 Limitations

This study compares the therapeutic effects of nine CCPPs and draws relevant conclusions. However, there are still some limitations here, including: (1) Interpretation and global relevance: All included trials were conducted in China, which limits generalisability to other populations, as genetic and environmental factors may influence drug efficacy; (2) Network geometry limitations: There is no closed loop between studies; NMA relies on indirect comparisons. Sparse connections, lack of closed loops, and absence of multi-arm trials may weaken the transitivity assumption and reduce the precision of indirect comparisons. (3) Diagnostic heterogeneity: Varying and sometimes non-validated definitions of CMD (e.g., symptom-based diagnosis, TTDE, variable CFR cut-offs) may introduce misclassification bias. (4) No adjustment for baseline covariates: Differences in patient characteristics, baseline CMD severity, and concomitant therapies were not accounted for in the NMA, potentially confounding results. (5) Potential publication bias: All included studies report positive effects; the absence of negative trials raises the possibility of reporting bias. (6) Evidence certainty: Given high/unclear risk of bias and lack of robust indirect evidence, the GRADE certainty for most outcomes is low; conclusions should be framed as hypothesis-generating rather than definitive. (7) Exclusion of international pharmacotherapy comparators: Standard CMD drugs (e.g., nicorandil, ranolazine, zibotentan) were not included in the network; therefore, the results cannot be directly compared to current international guideline-based treatments. (8) Research quality: Many trials have small sample sizes, increasing the risk of Type I/II errors and unstable SUCRA rankings. The absence of placebo-controlled and multicentre trials diminishes the robustness of the research findings.

## 5 Conclusion

This is among the first to evaluate the IMR and CFR to assess different CCPPs for CMD. The current NMA identified SXBX, XB, TXL, YXTL, XKS, and YDXNT as the most effective CCPPs for lowering IMR and LDL-C levels, improving CFR, reducing angina attack frequency, lowering hs-CRP levels, lowering ET-1 levels, and increasing NO levels. Moreover, the research emphasized the beneficial effects of CCPPs in CMD patients and further explored the possible mechanisms. Our research emphasizes that some CCPPS may have advantages in specific outcomes, but the results are hypothesis-generating and suggest some CCPPs may be associated with improvements in specific outcomes; confirmation in multicenter, head-to-head RCTs is needed.

## Data Availability

The original contributions presented in the study are included in the article/Supplementary Material, further inquiries can be directed to the corresponding authors.

## Abbreviations

CCPP, commercial Chinese polyherbal preparation; CMD, Coronary microvascular dysfunction; CAD, coronary artery disease; NMA, network meta-analysis; CNKI, China National Knowledge Infrastructure; VIP, China Science and Technology Journal Database; CBM, Chinese Biomedical Literature database; RCTs, randomized controlled trials; SXBX, Shexiangbaoxin Pill; TXL, Tongxinluo Capsule; SXTXD, Shexiangtongxindi Pill; YDXNT, Yindanxinnaotong Capsule; KDL, Kedalin Tablet; XB, Xinbao Pill; XKS, Xinkeshu Tablet; DAXXK, Diaoxinxuekang Capsule; YXTL, Yixintongluo Capsule; IMR, the Index of Microcirculatory Resistance; CFR, Coronary Flow Reserve; hs-CRP, hypersensitive C-reactive protein; ET-1, Endothelin-1; NO, Nitric oxide; LDL-C, Low-density lipoprotein cholesterol; CT, Conventional therapy; SUCRA, Surface under the cumulative ranking curve; RR, Risk ratio; MD, Mean difference; 95%CI, 95% confidence interval; ADRs, adverse drug reactions.
