# Supplementary material for: Comparative efficacy of commercial Chinese polyherbal preparation for coronary microvascular dysfunction: a systematic review and network meta-analysis of randomized controlled trials
Source: Front Pharmacol. 2025 Nov 4;16:1642864. doi: 10.3389/fphar.2025.1642864 (PMC12623403; doi:10.3389/fphar.2025.1642864)
Supplement: Supplementary file 1 [file Supplementaryfile1.pdf]

Supplementary Material

Supplement to:

Comparative efficacy of commercial Chinese polyherbal preparation for coronary microvascular dysfunction: a systematic review and network meta-analysis of randomized controlled trials

Wujiao Wang[1,†], Jun Zhang [2,†], Xinyue Wang[1,†], Yuxuan Li [1,3,†], Yudou Li[1,†], Fenglan Pu [4], Zhifei Yang [3], Jie Wan [3], Haiyan Zhu [1], Tianli Li [5,*], Peifen Chang[1,*]

Appendix 1. Study Protocol

1.1. Changes to the study protocol

Post-hoc sensitivity analyses: To evaluate transitivity,we agreed to perform two additional post-hoc sensitivity analyses of the primary outcomes: (1) exclusion of studies withhigh-risk; (2)exclusion of studies non-validated CMD. Which further demonstrating the robustness of our findings.

We conducted further meta-regression to identify sources of heterogeneity. Six characteristics were selected, including duration of intervention, CMD diagnosis methods, sample size, gender ratio, year of publication, and risk of bias. However, the regression analyses revealed no significant influence from these covariates, indicating that these characteristics were not sources of heterogeneity between studies.

Terminology: We Replaced "coronary microvascular disease (CMVD)" with coronary microvascular dysfunction (CMD)" throughout — CMD is the predominant term in contemporary literature and the preferred term in the 2024 ESC Guidelines on Chronic Coronary Syndromes. We Replaced " Chinese patent medicines" with "commercial Chinese polyherbal preparation (CCPP)", and Replaced " Western medicine" with "biomedicine" for scientific clarity incl.

Statistical analysis: We have added further description of how to assess transmissibility and consistency in the "Statistical analysis" section.

1.2. Study protocol

This study was registered with PROSPERO under registration number CRD42025632143. (https://www.crd.york.ac.uk/PROSPERO/)

Appendix 2. ConPhyMP checklists

2.1 ConPhyMP-checklists-table-1

ConPhyMP checklist of information for reporting plant material and its initial processing [1,2] (relevant for all studies on medicinal and food plants including extract types A, B, and C)

SECTION/TOPIC	ITEM NO.	CHECKLIST ITEM	YES	NO	NOT APPLICABLE	PAGE NO., IF ANY
Title and abstract	1	A clear and concise title including an informative abstract and balanced summary.	✓			
Description of the botanical drug and taxonomic authentication	2	Botanical or morphological authentication of the plant material (desirable is a combination with DNA barcoding, e.g., PCR, RFLP, genome sequencing) and the information must be included in a separate section of Material and Methods, if applicable, combined with the information required under item 3:	✓			
Description of the extract and extraction process	3	A separate section in Material and Methods, covers the relevant information on the material investigated, including the full species name(s), authorites and family; e.g. *Salvia miltorrhiza* Bunge [Lamiaceae; *Salviae miltorrhizae radix et rhizoma*], and on the processing and extraction of the crude drug including the traditional processing of the material used medicinally (fumigation, steaming, roasting, cooking, frying, etc.).	✓			
Documentation of the legal basis for collection and processing	4	Full compliance with the Nagoya protocol, CITES, and all associated treaties including phytosanitary regulations.	✓			
Description of product characteristics, in case of a finished (commercial) product	5	Information on the characteristics of the commercial products including batch number and date of production/best by information and regulatory status.	✓			

Note: Please also include here the following information about your submitted manuscript:

Name of the journal:

Date of the enquiry:

Title of the manuscript:

List of the authors:

Society for Medicinal Plant and Natural Product Research

Accessible at https://ga-online.org/conphymp

2.2 ConPhyMP-checklists-table-2a

ConPhyMP checklist of items for conducting and reporting analytical methods[1,2] relevant for extract type A (for species or botanical drugs covered in a monograph in one of the national or regional pharmacopoeias)

SECTION/TOPIC	ITEM NO.	CHECKLIST ITEM	YES	NO	NOT APPLICABLE	PAGE NO., IF ANY
Type of extract	1	A – Confirm that the species or botanical drug under investigation is covered in a monograph in one of the national or regional pharmacopoeias.	✓			
Preferred/main methods for extract characterisation/chemical analysis	2	Compliance with pharmacopoeial standards to be followed:				
		(a) The description of the active ingredients in the botanical drug (if known) or analytical marker compounds as defined.	✓			
		(b) An analysis as defined in the monograph is needed if the extract has not been supplied with a certificate.	✓			
		(c) If the preparation was purchased, the manufacturer and certificate of analysis need to be included.	✓			
		Including either the preferred or alternative approaches for characterisation:				
		(a) Triple chemical fingerprinting methods, each with one or more detection parameters.	✓	✓		
		(b) Quantification of at least two marker compounds (unless this is not feasible, evidence needs to be provided), and justification of the choice of markers (if applicable).	✓			
Alternative methods for extract characterisation/chemical analysis	3	(a) Single chemical fingerprinting method with at least three different detection parameters (i.e., altered detection parameters, like TLC/HPTLC with different staining reagents and/or UV excitation wavelengths, HPLC-DAD/LCDAD with different wavelengths). The same applies to coupling MS or NMR to chromatographic techniques.	✓			
		(b) Quantification of at least two marker compounds (unless this is not feasible, evidence needs to be provided), and justification of the choice of markers (if applicable).	✓			
Use of reference standards	4	(a) Direct overlay of the chromatogram of the sample with that of an officially specified reference standard (if applicable).<br>(b) Chromatographic fingerprinting: Direct overlay of the chromatogram of the sample with that of official reference standards of the powdered plant material or the dry extract from the plant material.	✓			
Comparison of different extracts/samples of the same plants	5	(a) Direct comparison of the chromatographic/spectroscopic system and/or scoring system for "similarity" to be followed.	✓			

Note: Please also include here the following information about your submitted manuscript:

Name of the journal:

Date of the enquiry:

Title of the manuscript:

List of the authors:

[1] Please acknowledge/cite this as follows: Heinrich M, Jalil B, Abdel-Tawab M, Echeverria J, Kulić Ž, McGaw LJ, et al. Best Practice in the chemical characterisation of extracts used in pharmacological and toxicological research—The ConPhyMP—Guidelines. Frontiers in Pharmacology. 2022;13:953205. https://doi.org/10.3389/fphar.2022.953205

[2] We strongly recommend reading this checklist in conjuncton with ConPhyMP 2022 explanaton and elaboraton for important clarifications on all items. If relevant, we also recommend after reading Heinrich et al. (2020) Best practice in research—Overcoming common challenges in phytopharmacological research. Journal of Ethnopharmacology. 2020;246:112230. https://doi.org/10.1016/j.jep.2019.112230

Appendix 3. Standard evaluation of commercial Chinese polyherbal preparations (CCPPs)

3.1 Table 1: Detail information of CCPPs

CCPPs	Source	Constituent(s)	Usage and dosage (Medicine instruction)	Quality control reported?	Chemical analysis reported? (Y/NR)
Shexiangbaoxin Pill	Shanghai Hehuang Pharmaceutical Co., Ltd	Moschus* [Moschidae; *Moschus berezovskii* Flerov, dried secretion], Ginseng Radix et Rhizoma [Araliaceae; *Panax ginseng* C.A.Mey., root and rhizome], Bovis calculus* [Bovidae; *Bos taurus* Linnaeus, gallstone], Cinnamomi Cortex* [Lauraceae; *Cinnamomum verum* J. Presl, bark], Styrax*[Altingiaceae; *Liquidambar orientalis* Mill., purified balsam], Bufonis Venenum* [Bufonidae; *Bufo bufo gargarizans* Cantor, dried secretion], Borneolum* [Lauraceae; *Cinnamomum camphora* (L.) J. Presl, synthetic product].	1-2 pills (22.5mg/pill), tid, po	Y-Prepared according to NMPA: Z31020068	NR
Tongxinluo Capsule	Shijiazhuang Ealing Pharmaceutical Co., Ltd.	Ginseng Radix et Rhizoma [Araliaceae; *Panax ginseng* C.A.Mey, root and rhizome], Scorpio* [Buthidae; *Buthus martensii* Karsch, dried body], Hirudo* [Hirudinidae; *Hirudo nipponica* Whitman, dried body], Eupolyphaga/Steleophaga* [Corydiidae; *Eupolyphaga sinensis* Walker, dried female body] ， Scolopendra* [Scolopendridae; *Scolopendra subspinipes mutilans* L. Koch, dried body], Cicadae Periostracum* [Cicadidae; *Cryptotympana pustulata* Fabricius, nymph exuviae], Paeoniae Radix Alba [Paeoniaceae; *Paeonia lactiflora* Pall., root], Borneolum* [Lauraceae; Synthetic product derived from *Cinnamomum camphora* (L.) J. Presl], Santalum Albi Lignum [Santalaceae; *Santalum album* L., heartwood], Dalbergiae Odoriferae Lignum [Fabaceae; *Dalbergia odorifera* T.C. Chen, heartwood], Olibanum [Burseraceae; *Boswellia carterii* Birdw., resin], Ziziphi Spinosae Semen [Rhamnaceae; *Ziziphus jujuba* var. *spinosa* (Bunge) Hu exH. F. Chow, seed]	2-4 capsules (0.26g/ capsule), tid, po	Y-Prepared according to NMPA: Z19980015	NR
Shexiangt	InnerMon	Moschus* [Moschidae; *Moschus berezovskii* Flerov, dried secretion], Ginseng Radix et Rhizoma [Araliaceae; *Panax ginseng* C. A. Mey., root and rhizome], Bufonis Venenum* [Bufonidae; *Bufo bufo gargarizans* Cantor, dried secretion], Salviae Miltiorrhizae Radix et Rhizoma[Lamiaceae; *Salvia miltiorrhiza*	2 pills (35mg/pill), tid, po	Y-Prepared according to NMPA: Z20080018	NR

ongxindi Pill	golia Kang En Bei Pharmaceutical Co., Ltd.	Bunge, rootand rhizome], Bovis Calculus* [Bovidae; *Bos taurus* Linnaeus, gallstone], Fel Ursi* [Ursidae; *Ursus thibetanus* Cuvier and/or *Ursus arctos* Linnaeus, gallbladder. The original trial report lacked specificationof the exact species], Borneolum* [Lauraceae; Synthetic productderived from *Cinnamomum camphora* (L.) J. Presl. The exact source material was not detailed in the report]			
Yindanxinnaotong Capsule	Guizhou Bailing Enterprise Group Pharmaceutical Co., Ltd.	Ginkgo Folium [Ginkgoaceae; *Ginkgo biloba* L., leaf], Salviae Miltiorrhizae Radix et Rhizoma [Lamiaceae; *Salvia miltiorrhiza* Bunge, root and rhizome], Erigerontis Herba [Asteraceae; *Erigeron breviscapus* (Vaniot) Hand.-Mazz., whole herb], Gynostemmatis Herba [Cucurbitaceae; *Gynostemma pentaphyllum* (Thunb.) Makino, whole plant], Crataegi Fructus [Rosaceae; *Crataegus pinnatifida* Bunge, fruit], Allii Sativi Bulbus [Liliaceae; *Alium sativum* L., bulb], Notoginseng Radix et Rhizoma [Araliaceae; *Panax notoginseng* (Burkill) F. H. Chen, root and rhizome], Blumeae Folium [Asteraceae; *Blumea balsamifera* (L.) DC., leaf]	2-4 capsules (0.4g/ capsule), tid, po	Y-Prepared according to NMPA: Z20027144	NR
Kedalin Tablet	Zhejiang Kang Enbei Pharmaceutical Co., Ltd.	Corydalis Rhizoma [Papaveraceae; *Corydalis yanhusuo* W. T. Wang, rhizome]	2-3 tablets (2.4mg/ capsule), tid, po	Y-Prepared according to NMPA: Z20044361	NR
Xinbao Pill	Guangdong Xinbao Pharmaceutical Technology Co., Ltd.	Daturae Flos [Solanaceae; *Datura metel* L, dried flower], Ginseng Radix et Rhizoma [Araliaceae; *Panax ginseng* C. A. Mey., root and rhizome], Cinnamomi Cortex [Lauraceae; *Cinnamomum verum* J. Presl, bark], Aconiti Lateralis Radix Praeparata [Ranunculaceae; *Aconitum carmichaelii* Debx., processed lateral root], Cervi Cornu Pantotrichum* [Cervidae; *Cervus nippon* Temminck and/or *Cervus elaphus* Linnaeus, unossified antler. The original source did not specify which species was used.], Borneolum* [Lauraceae; Synthetic borneolderived from *Cinnamomum camphora* (L.) J. Presl. The source material (fresh branches and leaves)is inferred from common preparation methods], Moschus* [Moschidae; *Moschus berezovskii* Flerov, dried secretion], Notoginseng Radix et Rhizoma [Araliaceae; *Panax notoginseng* (Burkill) F. H. Chen, root and rhizome], Bufonis Venenum* [Bufonidae; *Bufo*	2-6 pills (60mg/pill), tid, po	Y-Prepared according to NMPA: Z44021843	NR

		bufogargarizans Cantor, dried secretion]			
Xinkeshu Tablet	Shandong Wohua Pharmaceutical Technology Co., Ltd.	Salviae Miltiorrhizae Radix et Rhizoma[Lamiaceae; *Salvia miltiorrhiza* Bunge, root and rhizome], Puerariae Lobatae Radix [Fabaceae(Leguminosae); *Pueraria lobata* (Willd.) Ohwi, root], Notoginseng Radix et Rhizoma [Araliaceae; *Panax notoginseng* (Burkill) F. H. Chen, root and rhizome], Crataegi Fructus [Rosaceae; *Crataegus pinnatifida* Bunge, fruit], Aucklandiae Radix [Asteraceae; *Aucklandia lappa* Decne., root]	4 tablets (0.31g/ capsule), tid, po	Y-Prepared according to NMPA: Z37020042	NR
Diaoxinxuekang Capsule	Chengdu Dio Pharmaceutical Group Co., Ltd.	Dioscoreae Rhizoma [Dioscoreaceae; *Dioscorea panthaica* Prain et Burk. and/or *Dioscorea nipponica* Makino, rhizome. The original trial report did not specify the exact species used]	1-2 capsules (0.1g/ capsule), tid, po	Y-Prepared according to NMPA: Z20050616	NR
Yixintongluo Capsule	Lu Pharmaceutical Co., Ltd.	Astragali Radix [Fabaceae; *Astragalus membranaceus* (Fisch.) Bunge, root], Ginseng Radix et Rhizoma [Araliaceae; *Panax ginseng* C. A. Mey., root and rhizome], Ophiopogonis Radix [Asparagaceae; *Ophiopogon japonicus* (L. f.) Ker Gawl., root], Salviae Miltiorrhizae Radix et Rhizoma[Lamiaceae; *Salvia miltiorrhiza* Bunge, root and rhizome], Dalbergiae Odoriferae Lignum [Fabaceae; *Dalbergia odorifera* T. C. Chen, heartwood], Aurantii Fructus [Rutaceae; *Citrus aurantium* L., fruit], Chuanxiong Rhizoma [Apiaceae; *Ligusticum chuanxiong* Hort., rhizome], Poria [Polyporaceae; *Wolfiporia cocos* (F. A.Wolf) Ryvarden et Gilb., sclerotium], Pinelliae Rhizoma [Araceae; *Pinellia ternata* (Thunb.) Makino, tuber], Trichosanthis Pericarpium [Cucurbitaceae; *Trichosanthes kirilowii* Maxim., pericarp], Allii Macrostemonis Bulbus [Amaryllidaceae; *Allium macrostemon* Bunge, bulb], Citri Reticulatae Pericarpium [Rutaceae; *Citrus reticulata* Blanco, pericarp], Glycyrrhizae Radix et Rhizoma [Fabaceae; *Glycyrrhizaa uralensis* Fisch. ex DC., root and rhizome]	4 capsules (NR/ capsule), tid, po	Y-Prepared according to NMPA: ZBZ1789	NR

*: non-botanical drug, the names refer to the Chinese pharmacopoeia 2025.

3.2 Table 2: Extract and extraction process description of CCPPs

Drug name	Extract and extraction process description
Shexiang Baoxin pill	*Moschus*, *Ginseng Radix et Rhizoma*, *Bovis calculus*, *Cinnamomi Cortex*, *Bufonis Venenum*, and *Borneolum* are crushed together into fine powder. Take *Styrax*. and add proper amount of Chinese Baijiu to make pills, dry, then Shexiang Baoxin pill is ready.
Tongxinluo capsule	After washing *Scorpio, Hirudo, Eupolyphaga steleophaga, Scolopendra,* and *Cicadae periostracum*, place them in a drying oven and dry them at a low temperature. Then, break them into pieces and grind them together into a fine powder. Extract *Ginseng Radix et Rhizoma* twice with a 70% ethanol solution at six times the concentration, refluxing for 3 hours the first time and 2 hours the second time. Combine the extracts, recover the ethanol until no alcohol taste remains, and set aside the ginseng residue. Add *Paeonia Radix Alba* to the ginseng residue and water solution, add water equal to 9 times the total weight of the raw materials, and heat and simmer twice—the first time for 3 hours, the second time for 2 hours. Combine the decoction liquids and concentrate to a relative density of 1.20–1.25 g/cm³ (measured at 60°C) to form a transparent paste. Then add the *Ginseng Radix et Rhizoma* ethanol extract, mix thoroughly, and place in an oven to dry at 60–70°C. Grind into a fine powder. Oven to dry at 60-70°C. Grind into a fine powder. Mix the *Borneolum* with the animal-based medicine evenly, then mix with the extract powder of *Paeonia Radix Alba* and other ingredients. Fill the capsules and pack them into boxes.
Shexiangtongxindi Pill	Moisten *Bovis calculus, Ginseng Radix et Rhizoma, Salviae Miltiorrhizae Radix et Rhizoma*, and *Fel Ursi* with an appropriate amount of water. Moisten *Bufonis Venenum and Borneolum* separately with an appropriate amount of ethanol. Add *Moschus,* red iron oxide, black iron oxide, and Polysorbate 80 separately to melted polyethylene glycol 6000, mix thoroughly, and then drop the mixture into dimethyl silicone oil to form pellets. Dry them, prepare 1000 pellets, coat with a film coating, and the product is ready.
Yindanxinnaotong Capsule	Grind the *Blumeae Folium* into an excellent powder; extract garlic oil from *Allii Sativi Bulbus*; crush the *Notoginseng Radix et Rhizoma* into a coarse powder and set aside; Grind *Ginkgo Folium*, add dilute ethanol, heat and reflux extract twice, each time for 2 hours, combine the extracts, recover the ethanol and concentrate to an appropriate amount, add to a pre-treated macroporous adsorption resin column, elute sequentially with water and 80% ethanol, collect the corresponding eluates, recover the ethanol, dry under reduced pressure, and grind into an excellent powder; *Salviae Miltiorrhizae Radix et Rhizoma* was extracted twice with ethanol under reflux heating conditions, with each extraction lasting 1.5 hours. The extract was filtered, the filtrates were combined, the ethanol was recovered, and the mixture was concentrated, dried, and ground into a fine powder. The residue was decocted with water twice, each time for 2 hours, filtered, the filtrates combined, concentrated to a transparent paste with a relative density of 1.13–1.15 (50°C), ethanol added to achieve an ethanol content of 70%, mixed thoroughly, allowed to stand, ethanol recovered, concentrated, dried, and ground into an excellent powder; Take *Erigerontis Herba*. Boil twice with water, each time for 2 hours, filter, combine the filtrates, concentrate to a relative density of 1.12–1.15 (50°C), add ethanol to achieve an alcohol content of 70%, stir evenly, let stand, take the supernatant, recover the ethanol, concentrate, dry, and grind into a high-quality powder; Add *Gynostemmatis Herba*, *Crataegi Fructus*, and *Panax notoginseng* coarse powder to water and boil twice, each time for 2 hours. The mixture is filtered, the filtrates are combined, and the solution is

	concentrated to a relative density of 1.13–1.15 (50°C). Ethanol was added to achieve an ethanol content of 70%, and the mixture was stirred evenly. Let it settle, then take the supernatant. Recover the ethanol, concentrate, dry, and grind the powder into an excellent powder. Add the coarse powder of *Gynostemmatis Herba*, *Crataegi Fructus,* and *Notoginseng Radix et Rhizoma* to water and boil twice, each time for 2 hours. Filter the mixture, combine the filtrates, and concentrate the solution to a relative density of 1.13–1.15 (50°C). Add ethanol to achieve an ethanol content of 70% and stir thoroughly. After standing, take the supernatant. Recover the ethanol, concentrate, dry, and grind the powder into high-quality powder. Mix the above ultra-fine powder with garlic oil, 25g beeswax, 8.4g soybean phospholipids, and 220g vegetable oil thoroughly. Press the mixture into 1000 tablets.
Kedalin Tablet	Take *Corydalis Rhizoma*, grind it into coarse powder, and extract it three times with 80% ethanol using reflux heating, each time for 4 hours. Combine the extracts, filter them, and concentrate the filtrate under reduced pressure into a thick paste. Determine the alkaloid content, add an appropriate amount of excipients, prepare granules, compress them into tablets, coat them with sugar, and the product is obtained.
Xinbao Pill	Grind *Daturae Flos, Ginseng Radix et Rhizoma, Cinnamomi Cortex, Aconiti Lateralis Radix Praeparata, Cervi Cornu Pantotrichum, Borneolum, Moschus, Notoginseng Radix et Rhizoma, and Bufonis Venenum* into powder and add them to the CH-200 trough-type mixer. Add purified water at a ratio of 14% of the herbal powder weight to form a soft material of appropriate hardness. The soft material is processed in a GHL-3OD granulator to form uniformly sized granules. The granules are then fed into a YUJ-18BZ pill-making machine to produce wet pills. After rolling the wet pills into spheres, they are coated with talcum powder and medicinal charcoal in a BY-1000 sugar-coating machine, then dried in a fluidised bed granulator to obtain Xingbao pills.
Xinkeshu Tablet	Take *Notoginseng Radix et Rhizoma*, *Aucklandiae Radix*, and part of *Crataegi Fructus,* grind into a fine powder. The remaining *Crataegi Fructus* and *Puerariae Lobatae Radix* are soaked in 60% ethanol at room temperature for 30 minutes, then subjected to reflux extraction twice. The ethanol extracts are combined, the ethanol is recovered, and the mixture is set aside. *Salviae Miltiorrhizae Radix et Rhizoma* is decocted twice with water, the decoctions are combined, filtered, and the filtrate is mixed with the reserved solution, homogenised, concentrated to an appropriate volume, and combined with the fine powder to form granules. These are dried, compressed into 1000 tablets (small) or 500 tablets (large), coated with a film coating, and the product is obtained.
Diaoxinxuekang Capsule	Take the fine powder of total steroidal saponins extracted from the *Dioscoreae Rhizoma*, sieved through a 100-mesh screen, mix it evenly with the matrix, and heat it in a water bath at 60°C to 70°C for 15 minutes, stirring constantly until the total steroidal saponins dissolve, to obtain the medicinal solution. Weigh the capsule shell materials according to the specified weight ratio, then gelatinize at 65±5°C. Filter and vacuum the gel solution for approximately 30–40 minutes. Keep the gel solution warm and set aside. Press the medicinal solution into soft capsules, cool and dry in a rotating drum for 4–6 hours, then dry at 25°C and relative humidity of 25–30% for 18–24 hours. Wipe clean with 95% ethanol, then blow dry with cold air; the product is ready.
Yixintongluo Capsule	NR

Appendix 4. Search Strategy

Databases	Search strategies	Hit Counts
**Pubmed**<br><br>**(https://pubmed.ncbi.nlm.nih.gov/advanced/)**	#1 MeSH= Microvascular Angina<br><br>#2 Microvascular Angina OR Angina, Microvascular OR X Syndrome, Angina OR Angina X Syndrome OR Angina X Syndromes OR Syndrome, Angina X OR Syndrome X, Cardiac OR Syndrome X, Angina OR Angina Syndrome X OR Angina Syndrome Xs OR Syndrome Xs, Angina OR Angina Pectoris with Normal Coronary Arteriogram OR Cardiac Syndrome X OR Coronary microvascular disease OR coronary microcirculatory disorder OR coronary microcirculatory<br><br>#3 #1 OR #2<br><br>#4 All Fields =randomized controlled trial[Publication Type] OR randomized[Title/Abstract] OR placebo[Title/Abstract]<br><br>#5 medicine chinese traditional or chinese medicine or chinese drug or Herb or chinese herb or herbal medicine or medicine herbal or chinese herbal medicine or traditional chinese medicine or traditional chinese herb or zhong yi xue or chinese medicine traditional or Prescription or Formulae or Grain or Capsule or Injection or Decoction or Tablet or Pill or Soup<br><br>#6 #3 AND #4 AND #5	106
**Web of science**<br><br>**(https://www.webofscience.com/wos/alldb/advanced-search)**	#1 ((((((((((((((TS=(Microvascular Angina)) OR TS=(Angina, Microvascular)) OR TS=(X Syndrome, Angina)) OR TS=(Angina X Syndrome)) OR TS=(Angina X Syndromes)) OR TS=(Syndrome, Angina X)) OR TS=(Syndrome X, Cardiac)) OR TS=(Syndrome X, Angina)) OR TS=(Angina Syndrome X)) OR TS=(Angina Syndrome Xs)) OR TS=(Syndrome Xs, Angina)) OR TS=(Angina Pectoris with Normal Coronary Arteriogram)) OR TS=(Cardiac Syndrome X)) OR TS=(Coronary microvascular disease)) OR TS=(coronary microcirculatory disorder)) OR TS=(coronary microcirculatory)<br><br>#2 TS= (randomized controlled trial)OR TS=(randomized) OR TS=(placebo)<br><br>#3 TS=(Medicine, Chinese Traditional) OR TS=(Chinese medicine) OR TS=(Chinese drug) OR TS=(Herb) OR TS=(Chinese herb) OR TS=(Herbal medicine) OR TS=(Medicine, Herbal) OR TS=(Chinese herbal medicine) OR TS=(Traditional Chinese medicine) OR TS=(Traditional Chinese herb) OR TS=(Zhong Yi xue) OR TS=(Chinese Medicine, Traditional) OR TS=(Prescription) OR TS=(formulae) OR TS=(grain) OR TS=(capsule) OR TS=(injection) OR TS=(Decoction) OR TS=(Tablet) OR TS=(Pill) OR TS=(Soup)<br><br>#4 #1 AND #2 AND #3	73

**Embase**<br><br>**(https://www.embase.com)**	#1 (((((((((((((microvascular angina OR angina, microvascular OR x) AND ('syndrome,'/exp OR syndrome,) AND ('angina'/exp OR angina) OR 'angina'/exp OR angina) AND x AND ('syndrome'/exp OR syndrome) OR 'angina'/exp OR angina) AND x AND syndromes OR 'syndrome,'/exp OR syndrome,) AND ('angina'/exp OR angina) AND x OR 'syndrome'/exp OR syndrome) AND x, AND ('cardiac'/exp OR cardiac) OR 'syndrome'/exp OR syndrome) AND x, AND ('angina'/exp OR angina) OR 'angina'/exp OR angina) AND ('syndrome'/exp OR syndrome) AND x OR 'angina'/exp OR angina) AND ('syndrome'/exp OR syndrome) AND xs OR 'syndrome'/exp OR syndrome) AND xs, AND ('angina'/exp OR angina) OR 'angina'/exp OR angina) AND pectoris AND with AND normal coronary arteriogram OR 'cardiac'/exp OR cardiac) AND ('syndrome'/exp OR syndrome) AND x OR coronary) AND microvascular AND ('disease'/exp OR disease) OR coronary) AND microcirculatory AND ('disorder'/exp OR disorder) OR coronary) AND microcirculatory<br><br>#2 ((((((((((('medicine,'/exp OR medicine,) AND ('chinese'/exp OR chinese) AND traditional OR 'chinese'/exp OR chinese) AND ('medicine'/exp OR medicine) OR 'chinese'/exp OR chinese) AND ('drug'/exp OR drug) OR 'herb'/exp OR herb OR 'chinese'/exp OR chinese) AND ('herb'/exp OR herb) OR herbal) AND ('medicine'/exp OR medicine) OR 'medicine,'/exp OR medicine,) AND herbal OR 'chinese'/exp OR chinese) AND herbal AND ('medicine'/exp OR medicine) OR traditional) AND ('chinese'/exp OR chinese) AND ('medicine'/exp OR medicine) OR traditional) AND ('chinese'/exp OR chinese) AND ('herb'/exp OR herb) OR zhong) AND yi AND xue OR 'chinese'/exp OR chinese) AND ('medicine,'/exp OR medicine,) AND traditional OR 'prescription'/exp OR prescription OR formulae OR 'grain'/exp OR grain OR 'capsule'/exp OR capsule OR 'injection'/exp OR injection OR 'decoction'/exp OR decoction OR 'tablet'/exp OR tablet OR 'pill'/exp OR pill OR 'soup'/exp OR soup<br><br>#3 randomized AND controlled AND trial OR randomized OR placebo<br><br>#4 #1 AND #2 AND #3	30
**The Cochrane Library**<br><br>**(https://www.cochranelibrary.com/advanced-search)**	#1 MeSH descriptor: [Microvascular Angina] explode all trees<br><br>#2 Microvascular Angina OR Angina, Microvascular OR X Syndrome, Angina OR Angina X Syndrome OR Angina X Syndromes OR Syndrome, Angina X OR Syndrome X, Cardiac OR Syndrome X, Angina OR Angina Syndrome X OR Angina Syndrome Xs OR Syndrome Xs, Angina OR Angina Pectoris with Normal Coronary Arteriogram OR Cardiac Syndrome X OR Coronary microvascular disease<br><br>#3 #1 OR #2<br><br>#4 (Medicine, Chinese Traditional):ti,ab,kw OR (Chinese medicine):ti,ab,kw OR (Chinese drug):ti,ab,kw OR (Herb):ti,ab,kw OR (Herb):ti,ab,kw OR (Herb):ti,ab,kw OR (Chinese herb):ti,ab,kw OR (Herbal medicine):ti,ab,kw OR (Medicine, Herbal):ti,ab,kw OR (Chinese herbal medicine):ti,ab,kw OR (Traditional Chinese medicine):ti,ab,kw OR (Traditional Chinese herb):ti,ab,kw OR (Zhong Yi xue):ti,ab,kw OR	315

	(Chinese Medicine, Traditional):ti,ab,kw OR (Prescription or formulae):ti,ab,kw OR (grain):ti,ab,kw OR (capsule):ti,ab,kw OR (injection):ti,ab,kw OR (Decoction):ti,ab,kw OR (Tablet):ti,ab,kw OR (Pill):ti,ab,kw OR (Soup):ti,ab,kw<br><br>#5 #3 AND #4	
**CNKI**<br><br>**(https://www.cnki.net/)**	SU= (微血管心绞痛 OR X 综合征 OR 微血管功能异常 OR 冠状动脉微血管 OR 冠脉微循环障碍 OR 冠状动脉微循环）AND SU=(随机对照试验 OR 随机对照 OR RCT OR 随机)	579
**WanFang**<br><br>**(https://www.wanfangdata.com.cn/)**	主题:(微血管心绞痛 OR X 综合征 OR 微血管功能异常 OR 冠状动脉微血管 OR 冠脉微循环障碍 OR 冠状动脉微循环) AND 主题:(随机对照试验 OR 随机对照研究 OR RCT OR 随机) AND 主题:(中医 OR 中医药 OR 中药 OR 草药 OR 中草药 OR 中成药 OR 中西医 OR 方剂 OR 药方 OR 颗粒 OR 注射剂 OR 汤 OR 丸 OR 散 OR 丹 OR 胶囊 OR 片)	1782
**China Science and Technology Journal Database**<br><br>**(http://qikan.cqvip.com/index.html)**	M=(微血管心绞痛 OR X 综合征 OR 微血管功能异常 OR 冠状动脉微血管 OR 冠脉微循环障碍 OR 冠状动脉微循环) AND M=(中医 OR 中医药 OR 中药 OR 草药 OR 中草药 OR 中成药 OR 中西医 OR 方剂 OR 药方 OR 颗粒 OR 注射剂 OR 汤 OR 丸 OR 散 OR 丹 OR 胶囊 OR 片) AND R=(随机对照试验 OR 随机对照研究 OR RCT OR 随机)	128
**SinoMed**<br><br>**(http://www.sinomed.ac.cn/index.jsp)**	(("随机对照试验"[摘要] OR "Randomized Controlled Trial"[摘要]) OR "随机对照"[摘要] OR "随机"[摘要] OR "RCT"[摘要]) AND ("微血管心绞痛"[标题] OR "X 综合征"[标题] OR "微血管功能异常"[标题] OR "冠状动脉微血管"[标题] OR "冠脉微循环障碍"[标题] OR "冠状动脉微循环"[标题])	249
**the Chinese Clinical Trial Registry (http://www.chictr.org.cn/index.aspx)**	在“注册题目”检索框中，键入“微循环障碍”	33
**ClinicalTrials.gov (www.clinicaltrials.gov/)**	In the search box for "Condition/disease", type " Microvascular Angina"；	301

Appendix 5 Calculation Formulas for Numerical Data Conversion

Table S5.1. The sample size conversion formula is required in this study*

Purpose of using a formula	Formula	Alphabetic interpretation
Convert Standard Error To Standard Deviation	$SD = SE \times \sqrt{n}$	SD: Standard Deviation, SE: Standard Error, n: Sample Size
Merge Subgroup Data	$SD = \sqrt{\dfrac{(N_1 - 1)SD_1^2 + (N_2 - 1)SD_2^2 + \dfrac{N_1 N_2}{N_1 + N_2}(M_1^2 + M_2^2 - 2M_1 M_2)}{N_1 + N_2 - 1}}$<br>$N = N_1 + N_2$<br>$M = \dfrac{N_1 M_1 + N_2 M_2}{N_1 + N_2}$	N1: Sample size of subgroup A, M1: Mean of subgroup A, SD1: Standard deviation of subgroup A, N2: Sample size of subgroup B, M2: Mean of subgroup B, SD2: Standard deviation of subgroup B SD: Merge standard deviation N: Merge sample size M: Merge mean
Given the Baseline Difference: Objective To Find the Endpoint SD	$SD = \dfrac{2RSD_b + \sqrt{4R^2 SD_b^2 - 4(SD_b^2 - SD_d^2)}}{2}$	SDb: Standard deviation at baseline, SDd: Standard deviation of the difference, R: Correlation coefficient, assumed to be 0.9 in this study
Utilize the Confidence Interval Between Groups to Convert Standard Deviations	$SD = \dfrac{\sqrt{N_2 + N_1} \times (UL - LL)}{2TINV(0.05, N_2 + N_1 - 2)}$	N1: Sample size of the control group, N2: Sample size of the experimental group, UL: Upper limit of the confidence interval, LL: Lower limit of the confidence interval, TINV = Excel function for t-value

*The calculation formulas used in the study refer to the Cochrane Handbook for Systematic Reviews of Interventions. Reference: Higgins JPT, Thomas J, Chandler J, Cumpston M, Li T, Page MJ, Welch VA (editors). Cochrane Handbook for Systematic Reviews of Interventions version 6.4 (updated August 2023). Cochrane, 2023. Available from www.training.cochrane.org/handbook.

Appendix 6

6.1 Risk of bias summary.

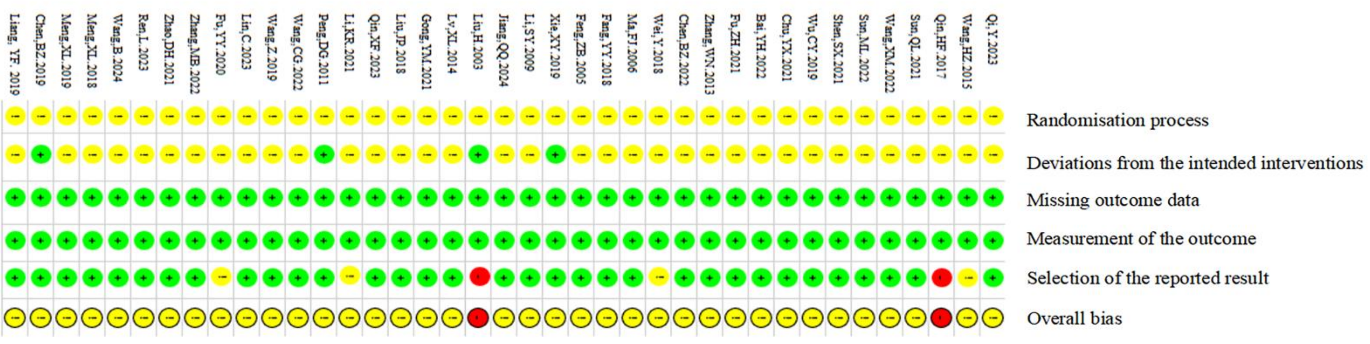

6.2 Risk of bias graph.

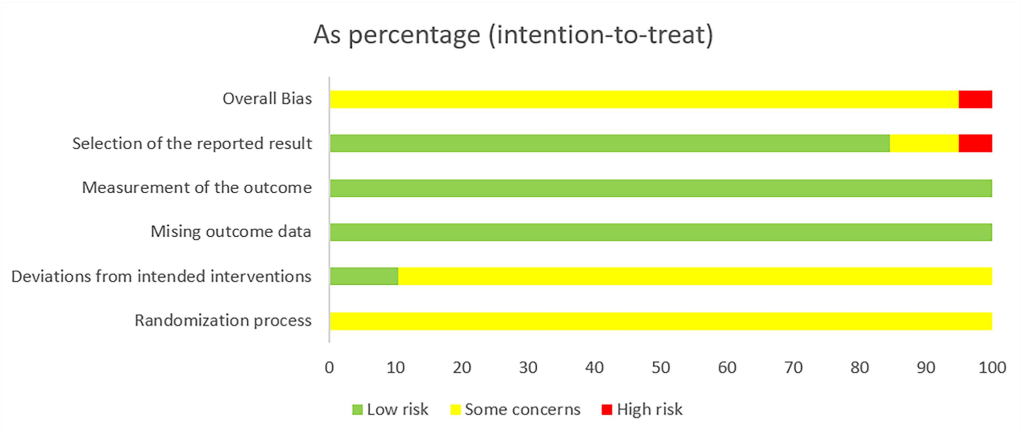

7 Forest plot

7.1 Forest plot of IMR

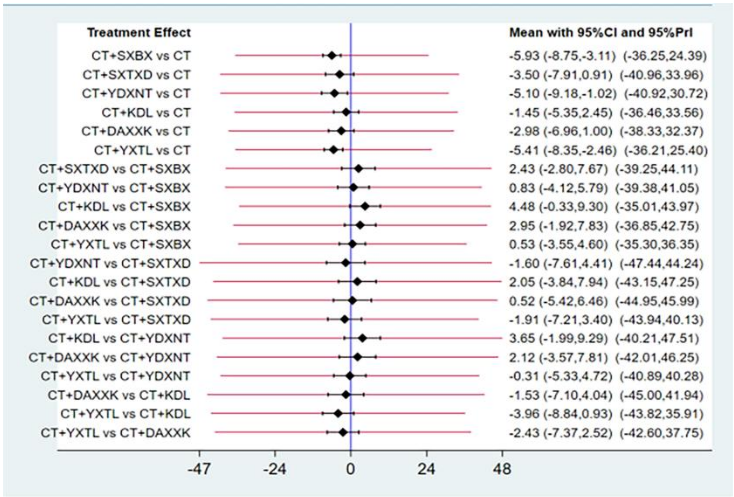

7.2 Forest plot of CFR

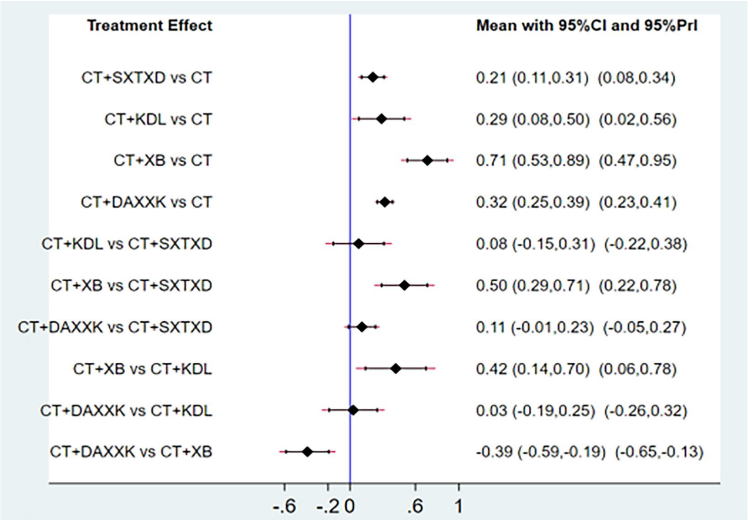

7.3 Forest plot of Angina attack frequency

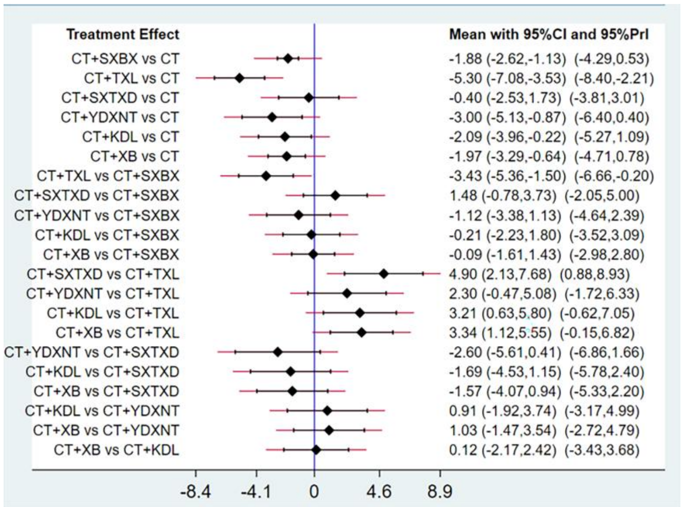

7.4 Forest plot of hs-CRP

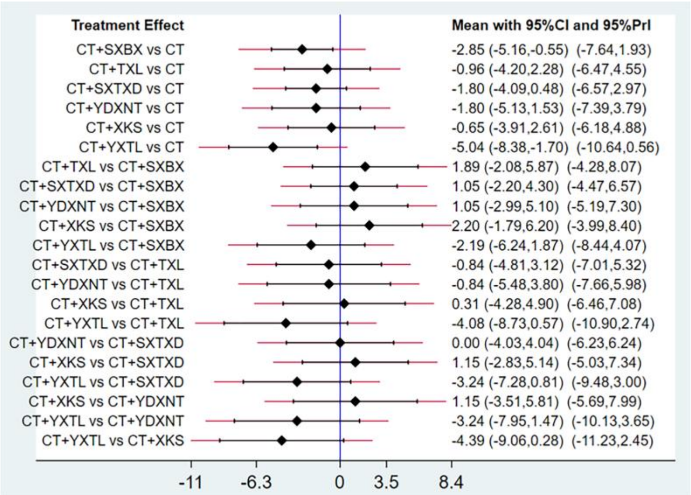

7.5 Forest plot of ET-1

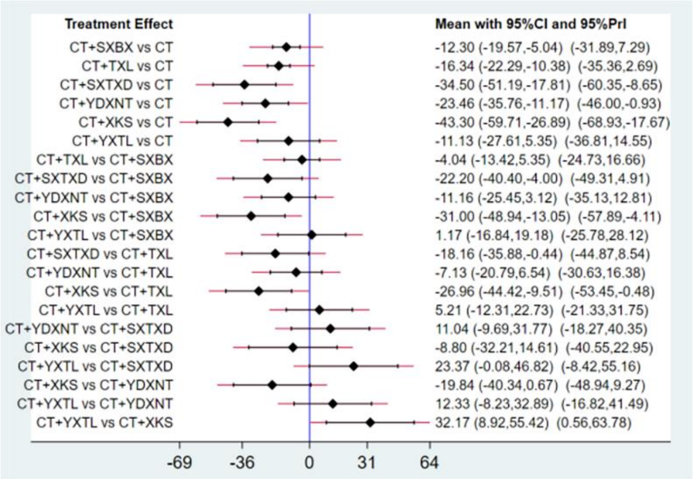

7.6 Forest plot of NO

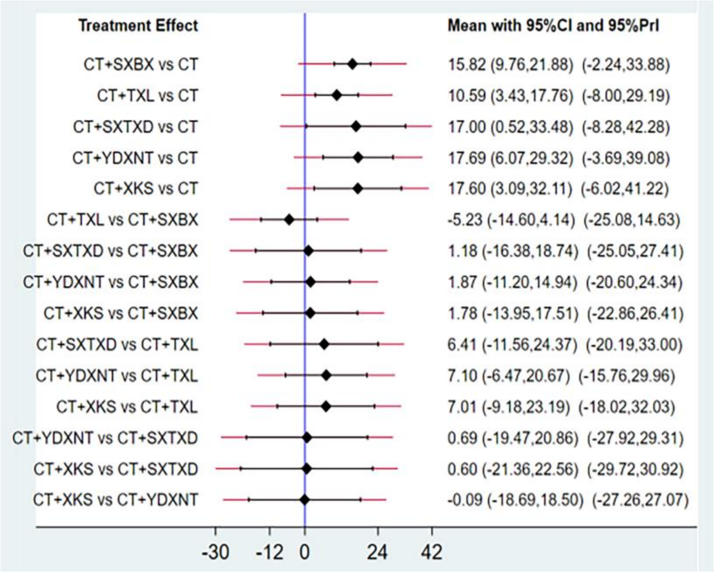

7.7 Forest plot of LDL-C

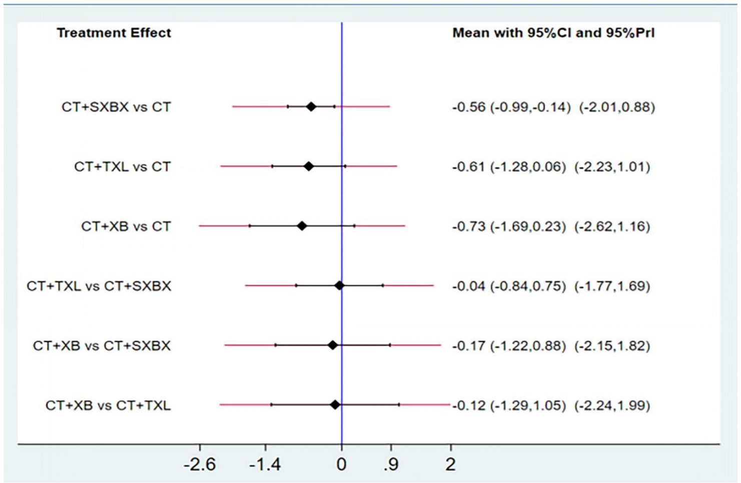

Appendix 8 The probability ranks of CCPP with different outcomes

Intervention	IMR		CFR		Angina attack frequency		hs-CRP	
	SUCRA (%)	Rank	SUCRA (%)	Rank	SUCRA (%)	Rank	SUCRA (%)	Rank
CT	6.3	7	0.1	5	6.3	7	13.7	7
CT+SXBX	81.8	1	-	-	48.3	5	70.9	2
CT+TXL	-	-	-	-	99.2	1	35.2	5
CT+SXTXD	50	4	32.1	4	17.3	6	53.2	3
CT+YDXNT	69.5	3	-	-	72.9	2	51.9	4
CT+KDL	25.3	6	53.7	3	53.6	3	-	-
CT+XB	-	-	99.9	1	52.4	4	-	-
CT+XKS	-	-	-	-	-	-	31.9	6
CT+DAXXK	41.7	5	64.2	2	-	-	-	-
CT+YXTL	75.5	2	-	-	-	-	93.2	1

Intervention	ET-1		NO		LDL-C	
	SUCRA (%)	Rank	SUCRA (%)	Rank	SUCRA (%)	Rank
CT	1.6	7	0.6	6	4.1	4
CT+SXBX	30.7	5	62	4	60.5	3
CT+TXL	45.1	4	34.6	5	63.6	2
CT+SXTXD	83.2	2	65.3	3	-	-
CT+YDXNT	64	3	70.4	1	-	-
CT+KDL	-	-	-	-	-	-
CT+XB	-	-	-	-	71.8	1
CT+XKS	96	1	67.1	2	-	-
CT+DAXXK	-	-	-	-	-	-
CT+YXTL	29.4	6	-	-	-	-

Appendix 9 Inconsistency, Heterogeneity, Transitivity assessment and meta-regression

9.1. Evaluation of Inconsistency

As this network meta-analysis does not form a closed loop, node splitting cannot be employed for inconsistency testing. we conducted a global consistency test.

Global Consistency.	I^2	*P* value
IMR	0.1327	87.07
CFR	0.7324	81.29
Angina attack frequency	0.0009	98.43
hs-CRP	0.2634	99.28
ET-1	0.00	99.56
NO	0.001	95.62
LDL-C	0.088	93.4

9.2 Evaluation of Heterogeneity of IMR

Treatment 1	Treatment 2	I^2 (%)	*P*	REML estimate of between-study variance (τ^2)
CT	CT+SXBX	94	=0.003	3.624
CT	CT+SXTXD	0.0	=0.0004	
CT	CT+YDXNT	0.0	<0.00001	
CT	CT+KDL	0.0	=0.01	
CT	CT+DAXXK	0.0	<0.0001	
CT	CT+YXTL	0.0	<0.0001	

9.3 Evaluation of Heterogeneity of CFR

Treatment 1	Treatment 2	I^2 (%)	*P*	τ^2
CT	CT+YDXNT	0.0	<0.0001	0.02
CT	CT+KDL	0.0	=0.0006	
CT	CT+XB	0.0	<0.00001	
CT	CT+DAXXK	0.0	<0.00001	

9.4 Evaluation of Heterogeneity of Angina attack frequency

Treatment 1	Treatment 2	I^2 (%)	*P*	τ^2
CT	CT+SXBX	98	<0.0001	0.893
CT	CT+TXL	0.0	<0.00001	
CT	CT+SXTXD	0.0	=0.46	

CT	CT+YDXNT	0.0	＜0.00001	
CT	CT+KDL	0.0	＜0.00001	
CT	CT+XB	99	=0.06	

9.5 Evaluation of Heterogeneity of hs-CRP

Treatment 1	Treatment 2	I^2 (%)	*P*	τ^2
CT	CT+SXBX	89	＜0.0001	2.706
CT	CT+TXL	0.0	＜0.00001	
CT	CT+SXTXD	100	=0.24	
CT	CT+YDXNT	0.0	＜0.0001	
CT	CT+XKS	0.0	=0.01	
CT	CT+YXTL	0.0	＜0.00001	

9.6 Evaluation of Heterogeneity of ET-1

Treatment 1	Treatment 2	I^2 (%)	*P*	τ^2
CT	CT+SXBX	100	=0.0006	65.47
CT	CT+TXL	98	=0.0009	
CT	CT+SXTXD	0.0	＜0.00001	
CT	CT+YDXNT	52	＜0.00001	
CT	CT+XKS	0.0	＜0.00001	
CT	CT+YXTL	0.0	＜0.00001	

9.7 Evaluation of Heterogeneity of NO

Treatment 1	Treatment 2	I^2 (%)	*P*	
CT	CT+SXBX	97	＜0.0001	54.19
CT	CT+TXL	77	＜0.0001	
CT	CT+SXTXD	0.0	＜0.00001	
CT	CT+YDXNT	93	=0.08	
CT	CT+XKS	0.0	＜0.00001	

9.8 Evaluation of Heterogeneity of LDL-C

Treatment 1	Treatment 2	I^2 (%)	*P*	
CT	CT+SXBX	95	=0.002	0.2239

CT	CT+TXL	47	＜0.00001
CT	CT+XB	0.0	＜0.00001

9.9 Transitivity assessment and meta-regression

_ES	Coef.	Std. Err.	t	P>|t|	[95% Conf.	Interval]
durationofintervention	-.0798025	2.387262	-0.03	0.979	-30.41284	30.25323
cmddiagnosis	1.096002	2.885792	0.38	0.769	-35.57146	37.76347
genderratio	2.315599	2.40096	0.96	0.512	-28.19149	32.82269
samplesize	2.553263	2.415663	1.06	0.482	-28.14065	33.24718
yearofpublication	1.999944	2.403144	0.83	0.558	-28.5349	32.53479
riskofbias	1.293524	3.433749	0.38	0.771	-42.33639	44.92344
_cons	-18.20779	8.290012	-2.20	0.272	-123.5424	87.1268

Appendix 10 sensitivity analysis

10.1 sensitivity analysis

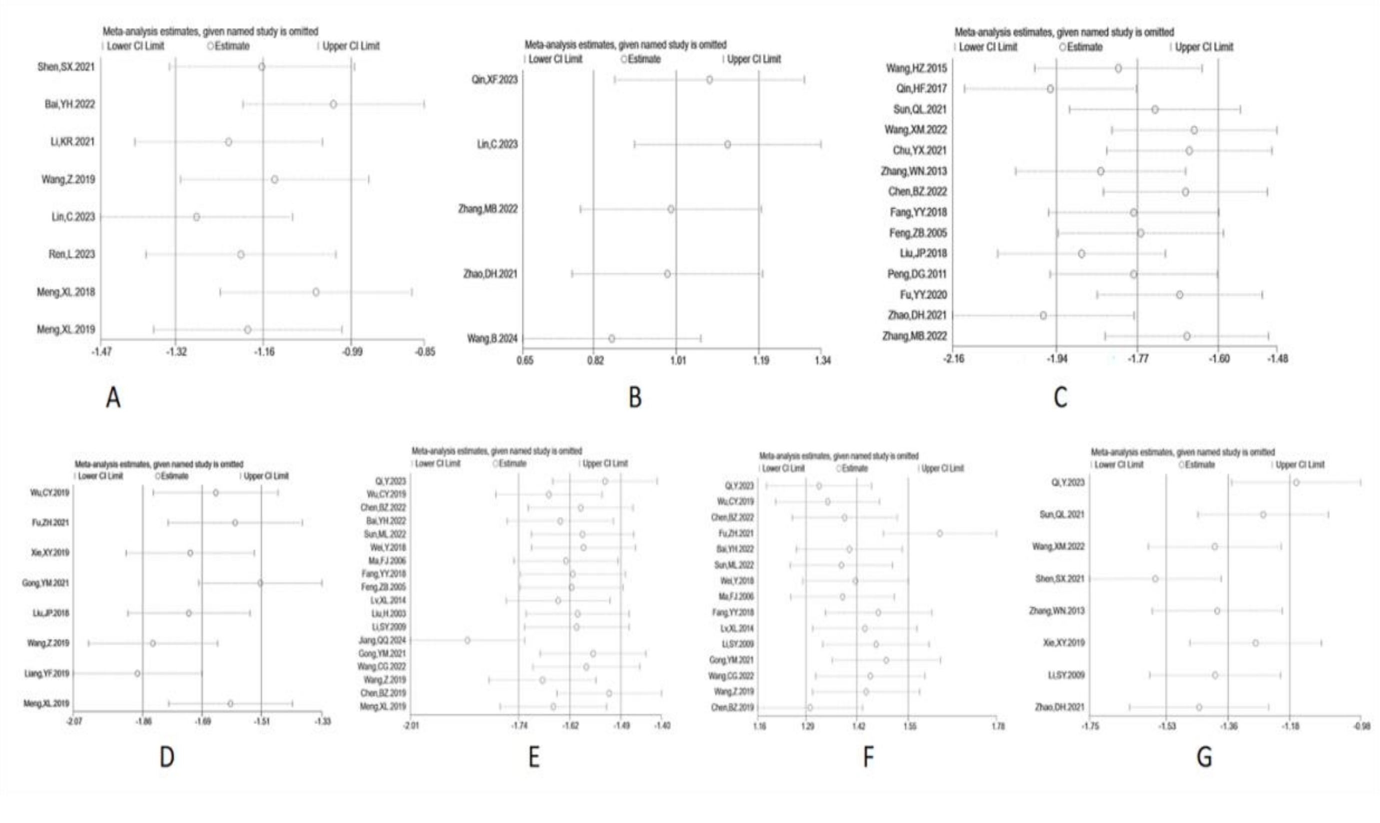

(A) IMR. (B) CFR. (C) Angina attack frequency. (D) hs-CRP. (E) ET-1. (F) NO. (G) LDL-C.

10.2 sensitivity analysis (excluding high- risk- of- bias)

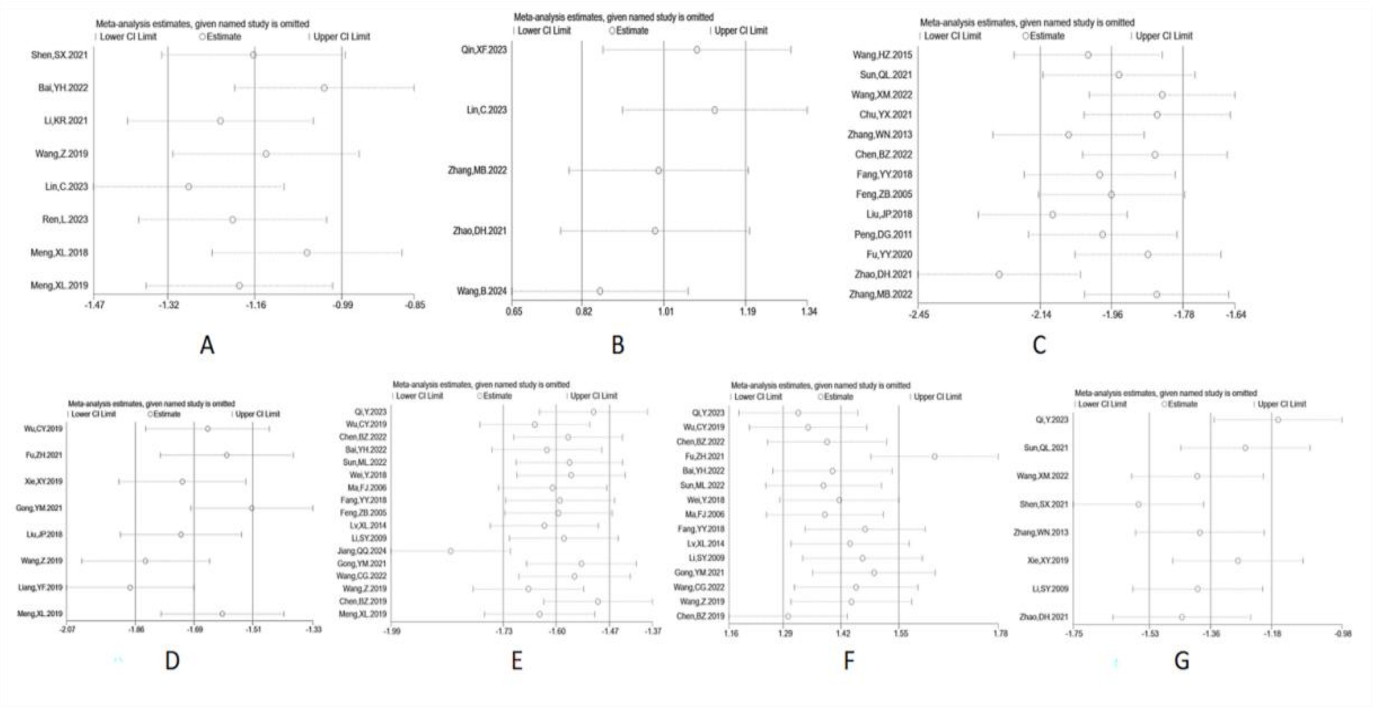

(A) IMR. (B) CFR. (C) Angina attack frequency. (D) hs-CRP. (E) ET-1. (F) NO. (G) LDL-C.

10.3 sensitivity analysis (excluding high- risk- of- bias)

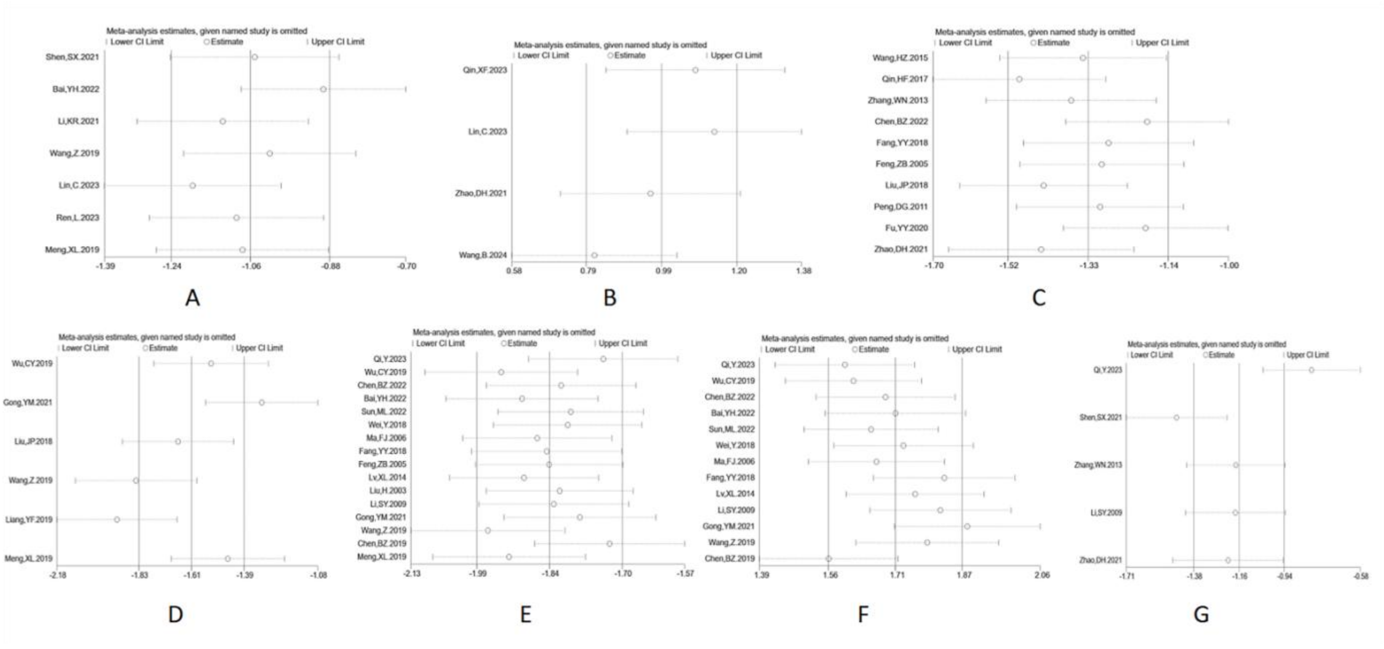

(A) IMR. (B) CFR. (C) Angina attack frequency. (D) hs-CRP. (E) ET-1. (F) NO. (G) LDL-C.

Appendix 11 Detailed data of Safety evaluation

All ADRs were mild, and no cases of withdrawal due to ADRs were reported.

Study ID	Sample (Intervention /Control group)	Intervention	Intervention group	Control group
Qi, Y. 2023	53/55	SXBX+CT	NR	NR
Wang, HZ. 2015	20/20	SXBX+CT	NR	NR
Qin, HF. 2017	30/30	SXBX+CT	(6/30)<br>5 Numbness of the tongue following sublingual administration, 1 abdominal distension	(1/30)<br>1 headache
Sun, QL. 2021	43/43	SXBX+CT	NR	NR
Wang, XM. 2022	40/40	SXBX+CT	0	0
Sun, ML. 2022	56/55	SXBX+CT	NR	NR
Shen, SX. 2021	32/32	SXBX+CT	NR	NR
Wu, CY. 2019	38/38	SXBX+CT	(2/38)<br>1 rash, 1 palpitations	(10/38)<br>2 headache, 3 rash, 2 loss of appetite, 3 palpitations
Chu, YX. 2021	37/37	SXBX+CT	NR	NR
Bai, YH. 2022	39/39	SXBX+CT	NR	NR
Fu, ZH. 2021	82/82	SXBX+CT	NR	NR
Zhang, WN. 2013	28/28	SXBX+CT	0	0
Chen, BZ. 2022	35/35	SXBX+CT	NR	NR

Wei, Y. 2018	30/32	TXL+CT	（1/30）<br>1 headache	(2/32)<br>2 headache
Ma, FJ. 2006	24/20	TXL+CT	NR	NR
Fang, YY. 2018	34/34	TXL+CT	NR	NR
Feng, ZB. 2005	16/16	TXL+CT	NR	NR
Xie, XY. 2019	45/45	TXL+CT	0	0
Li, SY. 2009	36/32	TXL+CT	NR	NR
Jiang, QQ. 2024	106/106	TXL+CT	NR	NR
Liu, H. 2003	19/18	TXL+CT	NR	NR
Lv, XL. 2014	19/19	TXL+CT	NR	NR
Gong, YM. 2021	54/52	SXTXD+CT	NR	NR
Liu, JP. 2018	20/18	SXTXD+CT	NR	NR
Qin, XF. 2023	55/56	SXTXD+CT	NR	NR
Li, KR. 2021	36/36	SXTXD+CT	NR	NR
Peng, DG. 2011	23/23	YDXNT+CT	NR	NR
Wang, CG. 2022	65/65	YDXNT+CT	(3/65)<br>1diarrhoea, 1 loss of appetite, 1Nausea and vomiting	(12/65)<br>1 palpitations, 4 diarrhoea, 3 loss of appetite, 4Nausea and vomiting
Wang, Z. 2019	43/44	YDXNT+CT	NR	NR
Lin, C. 2023	46/48	KDL+CT	0	0
Fu, YY. 2020	32/32	KDL+CT	0	0

Zhang, MB. 2022	40/40	XB+CT	NR	NR
Zhao, DH. 2021	61/61	XB+CT	(10/61)<br>2 headache, 3 dizziness, 2 Nausea, 2 diarrhoea, 1 fatigue	(11/61)<br>4 headache, 2 dizziness, 2Nausea, 1 diarrhoea, 2 fatigue
Ren, L. 2023	44/44	DAXXK+CT	(2/44)<br>1 loss of appetite, 1 Nausea and vomiting	(8/44)<br>2 Dizziness, 3 loss of appetite, 1 Nausea and vomiting, 2 fatigue
Wang, B. 2024	43/44	DAXXK+CT	NR	NR
Liang, YF. 2019	38/39	XKS+CT	NR	NR
Chen, BZ. 2019	60/60	XKS+CT	NR	NR
Meng, XL. 2018	60/60	YXTL+CT	NR	NR
Meng, XL. 2019	40/40	YXTL+CT	0	0

Appendix 12 Publication bias Funnel plots

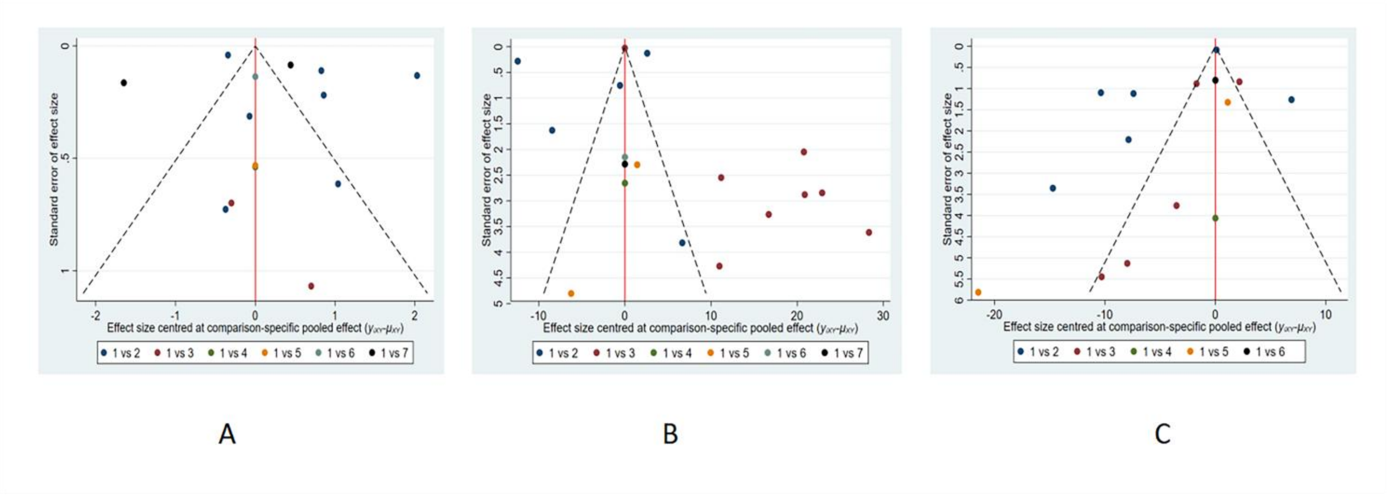

The funnel plot of (A) Angina attack frequency; (B) ET-1; (C) NO.
